# Dual-functional significance of ATM-mediated phosphorylation of spindle assembly checkpoint component Bub3 in mitosis and the DNA damage response

**DOI:** 10.1016/j.jbc.2022.101632

**Published:** 2022-01-25

**Authors:** Mingming Xiao, Siyue Zhang, Zhuang Liu, Yaqi Mo, Han Wang, Xu Zhao, Xue Yang, Rebecca J. Boohaker, Yang Chen, Yamei Han, Hong Liu, Bo Xu

**Affiliations:** 1 Chongqing Key Laboratory of Intelligent Oncology for Breast Cancer, Chongqing University Cancer Hospital and Chongqing University School of Medicine, Chongqing, China; 2 Department of Biochemistry and Molecular Biology, Key Laboratory of Breast Cancer Prevention and Therapy, Ministry of Education, Tianjin Medical University Cancer Institute and Hospital, National Clinical Research Center for Cancer, Key Laboratory of Cancer Prevention and Therapy, Tianjin, Tianjin’s Clinical Research Center for Cancer, Tianjin, China; 3 Department of Pancreatic Surgery, Fudan University Shanghai Cancer Center, Shanghai, China; 4 Department of Oncology, Southern Research Institute, Birmingham, Alabama, USA

**Keywords:** ATM, Bub3, mitosis, DNA damage response, phosphorylation, γ-H2AX, gamma-H2A histone family member X, APC/C, anaphase-promoting complex or cyclosome, ATM, ataxia–telangiectasia mutated, Bub1, benzimidazoles 1, Bub3, benzimidazoles 3, Ct, threshold cycle, DDR, DNA damage response, DSB, DNA double-strand break, H2A-Thr120, histone H2A at threonine 120, HA, hemagglutinin, IF, immunofluorescence, IR, ionizing radiation, NHEJ, nonhomologous end-joining, RT–qPCR, RT–quantitative PCR, SAC, spindle assembly checkpoint, Ser135, serine 135, SILAC, stable isotope labeling of amino acids in cell culture

## Abstract

Both the DNA damage response (DDR) and the mitotic checkpoint are critical for the maintenance of genomic stability. Among proteins involved in these processes, the ataxia–telangiectasia mutated (ATM) kinase is required for both activation of the DDR and the spindle assembly checkpoint (SAC). In mitosis without DNA damage, the enzymatic activity of ATM is enhanced; however, substrates of ATM in mitosis are unknown. Using stable isotope labeling of amino acids in cell culture mass spectrometry analysis, we identified a number of proteins that can potentially be phosphorylated by ATM during mitosis. This list is highly enriched in proteins involved in cell cycle regulation and the DDR. Among them, we further validated that ATM phosphorylated budding uninhibited by benzimidazoles 3 (Bub3), a major component of the SAC, on serine 135 (Ser135) both *in vitro* and *in vivo*. During mitosis, this phosphorylation promoted activation of another SAC component, benzimidazoles 1. Mutation of Bub3 Ser135 to alanine led to a defect in SAC activation. Furthermore, we found that ATM-mediated phosphorylation of Bub3 on Ser135 was also induced by ionizing radiation-induced DNA damage. However, this event resulted in independent signaling involving interaction with the Ku70–Ku80–DNA-PKcs sensor/kinase complex, leading to efficient nonhomologous end-joining repair. Taken together, we highlight the functional significance of the crosstalk between the kinetochore-oriented signal and double-strand break repair pathways *via* ATM phosphorylation of Bub3 on Ser135.

The DNA damage response (DDR) has a major impact on preventing genomic instability, which is one of the primary hallmarks of cancer (1, 2). Defects in the DDR not only contribute to cancer initiation and progression *via* mutation accumulation but also provide targetable vulnerabilities specific to cancer cells (3). DNA double-strand breaks (DSBs) are a highly toxic type of DNA damage that can occur throughout the cell cycle and lead to the passage of genetic alterations to daughter cells if they are not accurately repaired (4). DSBs are the primary cause of cell death induced by exposure to ionizing radiation (IR) (5). Once DSBs occur, the MRE11–NBS1–RAD50 complex is recruited to the damaged sites to maintain the DSB ends tethered to each other for repair (6). Thereafter, the ataxia–telangiectasia mutated (ATM) kinase, a member of the phosphatidylinositol-3 kinase-like family, is activated to phosphorylate a large number of proteins to execute the optimal DDR, including cell cycle checkpoints and programmed cell death (7, 8). Patients lacking functional ATM present multiple clinical features, such as progressive cerebellar ataxia, susceptibility to malignancies, variable immunodeficiency, hypersensitivity to IR, and increased incidence of metabolic diseases (9), indicating the critical role of ATM in genome stability.

The spindle assembly checkpoint (SAC) is another surveillance mechanism that maintains genomic stability by ensuring the fidelity of chromosome segregation during mitosis. Failure in the SAC leads to premature sister chromatid separation and aneuploidy (10). The SAC defect is involved in numerous human pathogeneses, including cancer formation. In addition to its role in the DDR, we previously reported that ATM is activated during mitosis through Aurora-B mediated serine 1403 phosphorylation (11). Several SAC proteins have been identified as ATM substrates, such as budding uninhibited by benzimidazoles 1 (Bub1), Mad1, Mad2BP, and Sgo1 (12), providing evidence that ATM plays an important role during mitosis. For example, ATM phosphorylation of Bub1 on Ser314 is required for Bub1 activity and the activation of the SAC (11). Mad1, when phosphorylated by ATM during mitosis, is required for its interaction with Mad2 (13). Despite these findings, the global profile of ATM substrates during mitosis is unknown.

Among the SAC proteins, Bub3 forms an inhibiting complex with Mad2 and Mad3/BubR1, which can block the anaphase-promoting complex or cyclosome (APC/C) by phosphorylating its coactivator Cdc20. After all chromosomes attach to microtubules, activation of APC/C triggers the transition from metaphase to anaphase during mitosis (14). In case of mitotic cells encountering DNA damage, mediator of DNA damage checkpoint 1 localizes to mitotic kinetochores after ATM phosphorylation of gamma-H2A histone family member X (γ-H2AX) (15). Thereafter, mediator of DNA damage checkpoint 1 binds to the mitotic checkpoint complex, Mad2, and Cdc20, and ATM is required for SAC activation. During mitosis, DSBs are mainly sensed by the MRE11–NBS1–RAD50 complex, boosting the recruitment of the Polo kinase. Polo kinase activity facilitates subsequent accumulation of the BubR1–Bub3 complex at the DSBs, where Bub3 and BubR1 depend on each other to localize laser-induced DNA lesions (16, 17). Apart from Bub3 being functional in response to DNA damage, a detailed mechanism for Bub3 in the DDR is largely unknown, and the crosstalk between the DDR and SAC remains to be elucidated.

In this study, we identified a cell cycle and DDR-enriched substrate list of mitotically activated ATM *via* stable isotope labeling of amino acids in cell culture (SILAC) mass spectrometry. Furthermore, we demonstrate that ATM phosphorylates Bub3 on serine 135 (Ser135) to activate the SAC and the DNA repair *via* independent pathways. The dual-functional role of ATM-mediated Bub3 Ser135 phosphorylation provides new insights into the interaction between the SAC and the DDR.

## Results

### Identification of mitotically activated ATM substrates by quantitative phosphorproteomics

Previous studies have demonstrated that ATM phosphorylates specific substrates in response to IR-induced DNA damage (18). However, the substrates for mitotically activated ATM are unknown. To achieve a comprehensive understanding of ATM substrate phosphorylation during mitosis, we employed the SILAC assay in combination with mass spectrometry (Fig. S1*A*). We detected 595 proteins that had undergone phosphorylation changes during mitosis (Table S1). Foremost was a subset of 18 proteins strongly associated with cell cycle regulation and DDR (Table 1 and Fig. S1*B*). These findings provide the first picture of an ATM-mediated global phosphorylation network during mitosis.

**Table 1:** Putative ATM substrates in mitosis by SILAC mass spectrometry

Protein	Function	ATM S/TQ sequence
Mitotic checkpoint protein BUB3	Spindle assembly checkpoint	Yes S135
Zinc finger protein 207	Spindle assembly checkpoint	Yes S104
X-ray repair crosscomplementing protein 6	DNA strand breaks repair	Yes S51
Non-POU domain–containing octamer-binding protein	DNA repair pathway	Yes S50
Serine/arginine-rich splicing factor of 5	RAN splicing regulator of cell cycle	Yes S239
Replication protein A 32kD subunit	Mitotic G1 damage checkpoint	Yes S174
PEST proteolytic signal–containing nuclear protein	Cell cycle regulation	Yes S14
RuvB-like 2	Chromatin remodeling replicative senescence	Yes S43
Protein SEC13 homolog	Component of nuclear pore complex	Yes S235
Transcriptional activator protein Pur-alpha	DNA replication initiation	Yes S127
RuvB-like 1	chromatin remodeling DNA replication	Yes T12
Serine/threonine-protein phosphatase PP1-alpha catalytic subunit	Regulates NEK2	Yes S48
Eukaryotic translation initiation factor 4 gamma 2	Cell cycle arrest/translation initiation	Yes S443
Ras GTPase-activating-like protein IQGAP1	Binds cdc42 cytoskeleton remodeling	Yes S1104
Splicing factor, praline rich and glutamine-rich	DNA repair pathway	Yes S392
Transcription elongation factor SPT5	Cell cycle	Yes S299

### Bub3 is phosphorylated on Ser135 in an ATM-dependent manner in mitosis

**Figure 1:**
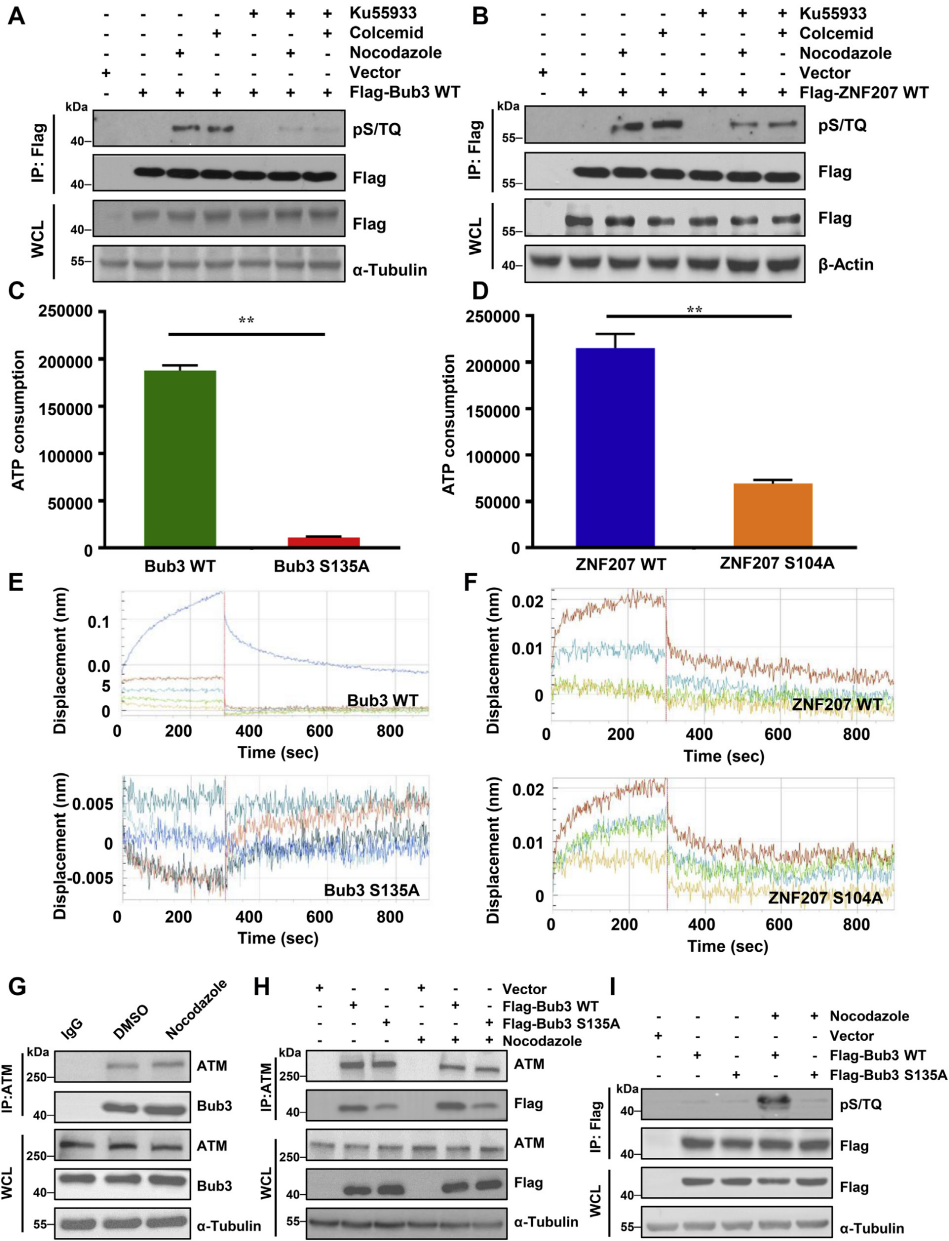
**Bub3 is phosphorylated on Ser135 in an ATM-dependent manner in mitosis.***A* and *B*, anti-FLAG immunoprecipitation followed by Western blot with the antiphosphor-S/TQ antibody. Cells transfected with vector, FLAG-Bub3 WT, or FLAG-ZNF207 WT were treated with or without nocodazole, colcemid, in the presence or the absence of the ATM inhibitor Ku55933. *C* and *D*, ATM kinase activity was quantified by the *in vitro* kinase assay followed by measuring the production of ADP. *E* and *F*, *in vitro* interaction of ATM and Bub3 or ZNF207 assessed by biolayer interferometry (BLI). *G*, HeLa cells treated with DMSO or nocodazole were immunoprecipitated with an anti-ATM antibody and analyzed by Western blot. *H*, HeLa cells transfected with vector only, FLAG-Bub3 WT, or FLAG-Bub3 S135A were treated with DMSO or nocodazole before immunoprecipitation with an anti-ATM antibody and Western blot with indicated antibodies. *I*, HeLa cells transfected with vector, FLAG-Bub3 WT, or FLAG-Bub3 S135A were treated with or without nocodazole. Immunoprecipitation of FLAG-tagged proteins was conducted followed by Western blot with the indicated antibodies. ATM, ataxia–telangiectasia mutated; Bub3, benzimidazoles 3; DMSO, dimethyl sulfoxide; Ser135, serine 135.

The top two mitotic-related proteins on the list are Bub3 and ZNF207, two components of the SAC. The corresponding phosphorylation sites were Ser135 for Bub3 and serine 104 for ZNF207 (Table 1). Multiple sequence alignment revealed well conservation of Bub3 (S/T135) or ZNF207 (S/T104) and flanking ATM substrate motifs in vertebrate (Fig. S2, *A* and *B*). To assess whether phosphorylation occurs in cells, we first immunoprecipitated FLAG-tagged Bub3 or ZNF207 and incubated it with the phosphor-S/T-Q antibody. We found that nocodazole or colcemid induced Bub3 S/TQ phosphorylation, which was significantly inhibited by the ATM inhibitor KU55933 (Fig. 1*A*). However, nocodazole or colcemid-induced S/TQ phosphorylation of ZNF207 was partly diminished by ATM inhibitor (Fig. 1*B*). In order to assess whether these phosphorylation events are not caused by DNA damage because of the exposure to colcemid and nocodazole, we analyzed γ-H2AX Western blotting in HeLa cells treated with mock, colcemid (20 ng/ml,17 h), nocodazole (200 nM,17 h), or IR (2 Gy, 2 h). We observed that the dose and exposure time of nocodazole we used did not induce γ-H2AX, compared with cells treated with IR (Fig. S2*C*). This experiment supports the notion that nocodazole-induced S/T-Q phosphorylation in Bub3 and ZNF207 is not because of DNA damage caused by nocodazole exposure. Colcemid, on the other hand, had a slight increase on the γ-H2AX level. For this reason, in the subsequent experiments, we used solely nocodazole for the mitotic response. Taken together, these data imply that Bub3 S/TQ phosphorylation is in an ATM-dependent manner in mitosis. In contrast, ZNF207 phosphorylation is partially dependent on ATM.

To test for direct phosphorylation, we performed the *in vitro* kinase assay, in which recombinant ATM was incubated with the Bub3 WT or Bub3 S135A peptides or the ZNF207 WT or ZNF207 S104A peptides in the ATM kinase buffer. We observed a strong kinase reaction with the WT peptide, whereas alanine substitution at the serine site caused a dramatic reduction in the phosphorylation signal. Noticeably, the phosphorylation signal completely disappeared in the Bub3 S135A group (Fig. 1*C*). However, alanine substitution at the serine site of ZNF207 was partly diminished in the phosphorylation signal (Fig. 1*D*).

Protein phosphorylation requires protein–protein interactions. To test whether Bub3 or ZNF207 binds to ATM, we performed the biolayer interferometry assay, which was used to characterize direct interactions in the *in vitro* system. We mutated the corresponding serine residues to alanines, in which alanine substitution would abolish phosphorylation. In this assay, increasing concentrations of WT or S135A of Bub3 peptides or WT or S104A of ZNF207 peptides (each 10 amino acids in length) were incubated with the full-length ATM. Based on the parameters from the condition optimization results, ATM-coated sensors and Bub3 WT or S135A and ZNF207 WT or S104A analytes were analyzed for binding on the Octet system. Compared with the WT peptide, the results confirmed that a significant number of Bub3 S135A showed decreased sensorgram patterns (Fig. 1*E*), indicating that (1) Bub3 is capable of binding to ATM and (2) S135A diminishes its binding. However, no binding signals were observed in either the ZNF207 WT or the S104A mutant peptides (Fig. 1*F*), suggesting that ZNF207 might not be preferable as an ATM substrate. Hence, between Bub3 Ser135 and ZNF207 serine 104, we mainly focused on Bub3 Ser135 phosphorylation for further functional studies.

To confirm Bub3 was an ATM substrate *in vivo*, we first performed coimmunoprecipitation experiments to assess the interaction between Bub3 and ATM. In cells expressing FLAG-tagged Bub3, we detected ATM in FLAG immunoprecipitates (Fig. S2*D*). In a reciprocal experiment, we also observed FLAG-Bub3 in ATM immunoprecipitates (Fig. S2*E*). Similarly, endogenous interactions of ATM and Bub3 were also observed (Fig. 1*G*). Furthermore, nocodazole exposure increased the interaction between Bub3 and ATM (Fig. 1*G*). We also investigated whether the S135A mutation affected its interaction with ATM in the presence or the absence of nocodazole. As shown in Figure 1*H*, compared with Bub3 WT, the interaction between Bub3 S135A and ATM was significantly decreased. To further assess whether Ser135 phosphorylation occurred in cells, we used the phosphor-S/T-Q antibody. We found that Bub3 S/TQ phosphorylation was induced by nocodazole, whereas the S135A mutation markedly diminished the phosphorylation signal (Fig. 1*I*). These results prove that Ser135 of Bub3 is phosphorylated by the ATM kinase *in vivo*.

### Overexpressing the Bub3 S135A mutant causes defects in the SAC

**Figure 2:**
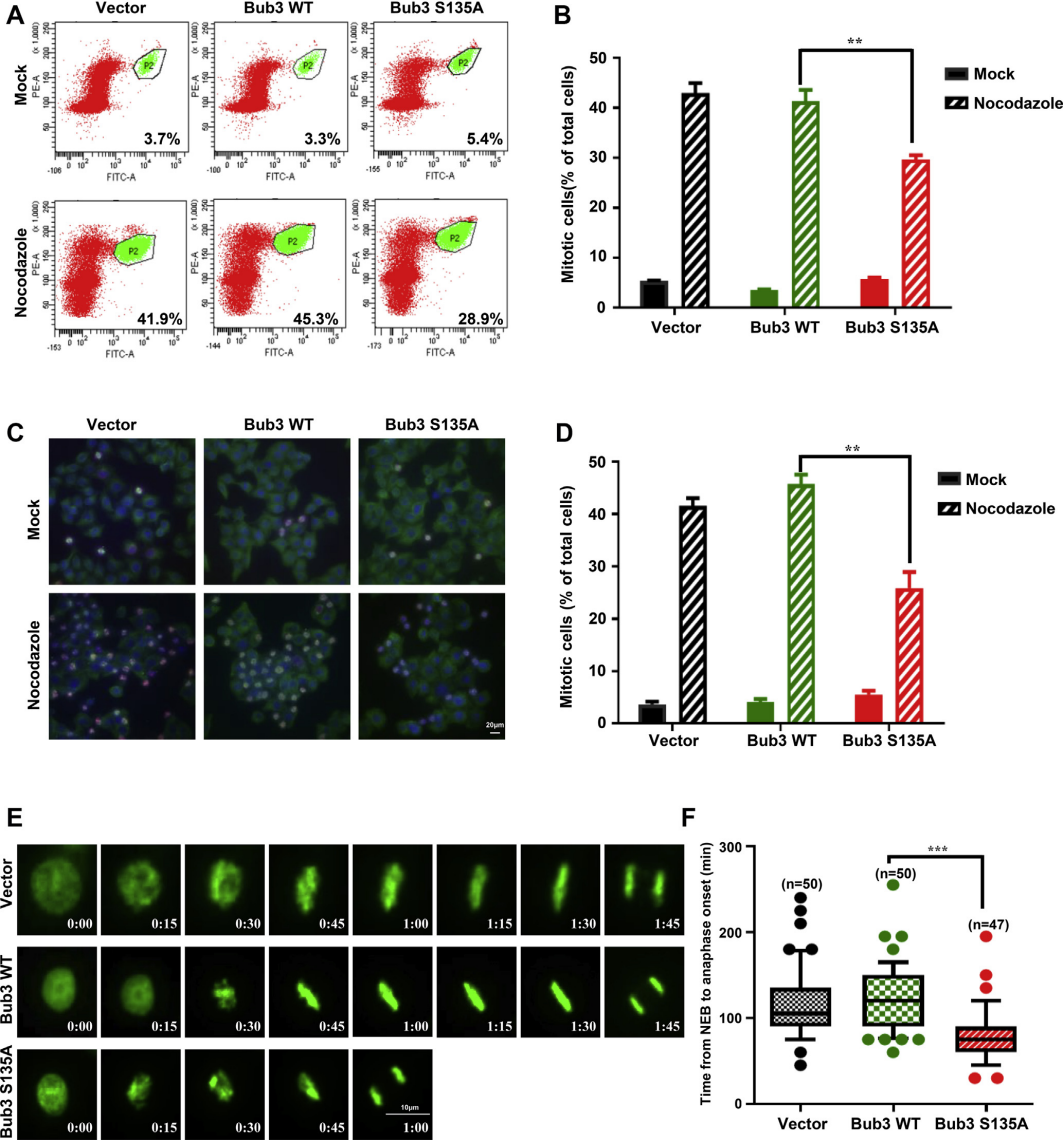
**Overexpression of Bub3 S135A mutation causes dysfunction of the SAC.***A*, HeLa cells transfected with vector, Bub3 WT, or S135A mutant were treated with nocodazole for 17 h and stained with a flow cytometry–based antiphosphor-histone-H3-Ser10 antibody to determine the mitotic index. *B*, quantification of the data in *A*. Error bars represent ±1 standard deviation, and the means of results from three independent experiments are graphed. *C*, HeLa cells transfected with vector, WT, or S135A were stained with α-tubulin and DAPI to visualize microtubules (*green*) and chromosomes (*blue*), respectively. *D*, quantitative data in (*C*) were collected from 100 cells for each group. The error bars represent ±1 standard deviation, and the means of results from three independent experiments are graphed. *E*, vector, Bub3 WT, and Bub3 S135A cells expressing H2B-GFP were imaged at the onset of mitosis to monitor chromosomal dynamics. Representative fluorescence images are shown. *F*, the average time from nuclear envelope breakdown (NEB) to anaphase onset in cell lines expressing vector, Bub3 WT, and Bub3 S135A was measured by time-lapse microscopy. Bub3, benzimidazoles 3; DAPI, 4′,6-diamidino-2-phenylindole; SAC, spindle assembly checkpoint.

To study the functional significance of Bub3 Ser135 phosphorylation, we first examined whether overexpression of the WT or S135A mutant of Bub3 could influence SAC activation. We transfected the vector, FLAG-tagged WT, or S135A Bub3 plasmid into HeLa cells. Compared with the vector or WT cells, the expression of S135A displayed a significant reduction in mitotic arrest, as measured by flow cytometry for histone-H3-serine 10 phosphorylation in response to nocodazole treatment (Fig. 2, *A* and *B*). In addition, we also found that cells exposed with the ATM inhibitor Ku55933 failed to arrest in mitosis after nocodazole treatment (Fig. S3, *A* and *B*). We also quantified the mitotic cells by immunofluorescence (IF) staining with the antiphosphor histone H3 serine 10 and α-tubulin antibodies. Consistent with the results of the aforementioned studies, the S135A mutation caused a significantly reduced mitotic arrest (Fig. 2, *C* and *D*). We also monitored the chromosome dynamics and timing of mitosis by time-lapse microscopy. We found that Bub3 S135A-expressing cells exited mitosis much earlier than Bub3 WT and vector cells (Fig. 2*E*). The time between nuclear envelope breakdown and anaphase onset was 117.9 ± 40.6 min (n = 50) in vector cells and 120.3 ± 36.9 min (n = 50) in Bub3 WT cells. However, in Bub3 S135A cells, the time had only 78.2 ± 32.3 min (n = 47) (Fig. 2*F*). These results suggest that Bub3 Ser135 phosphorylation is critical for the activation of the spindle checkpoint.

### Expression of Bub3 S135A causes chromosomal instability

**Figure 3:**
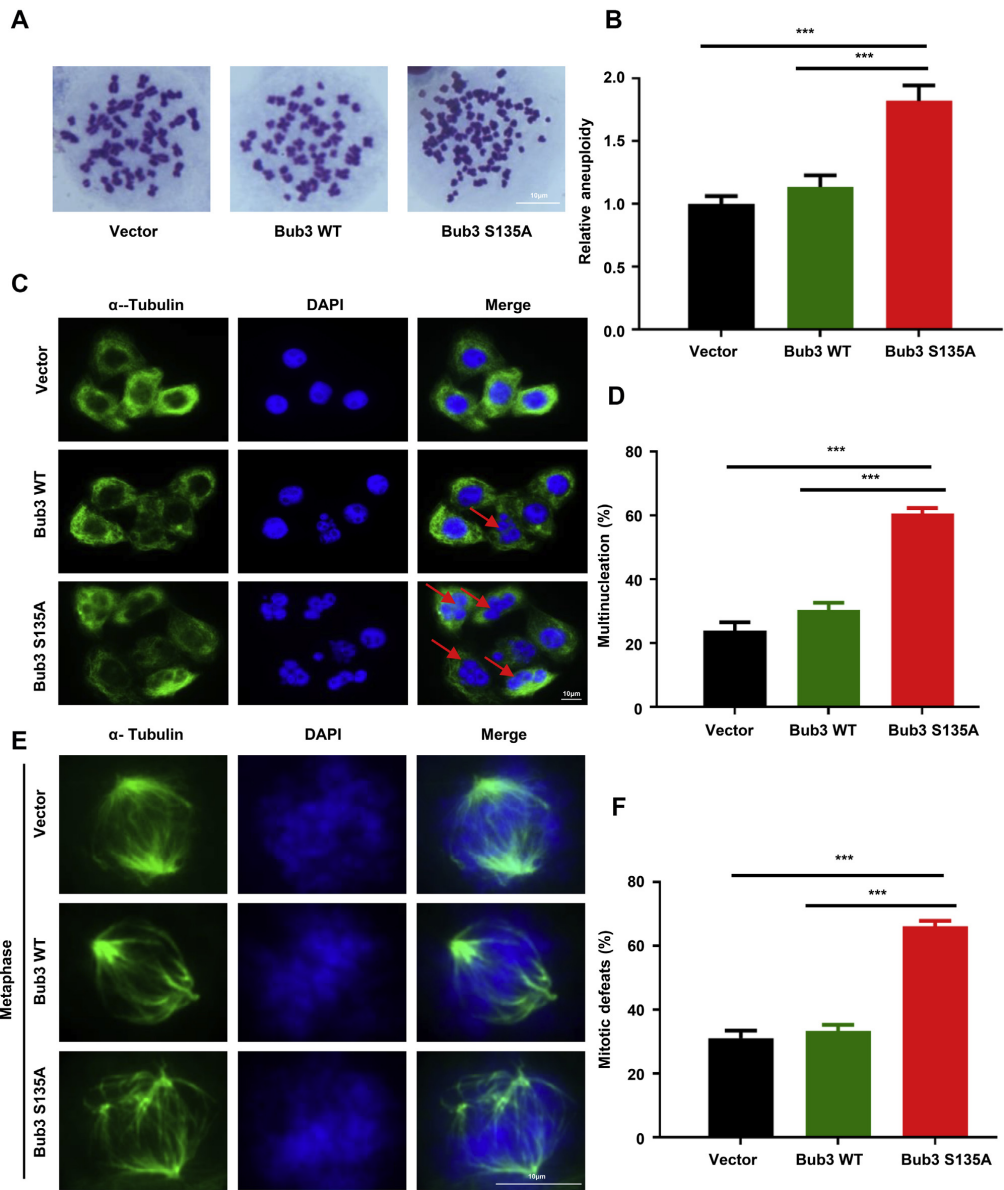
**Expression of Bub3 S135A causes chromosomal instability.***A*, the chromosome spread assay in HCT116 cells expressing vector, WT, or S135A Bub3. *B*, quantification of percentage of aneuploidy cells. Statistical analyses were conducted using *t* test; *p* values are presented. *C*, overexpression of Bub3 S135A resulted in multinucleated cells. Representative photos of vector, FLAG-tagged Bub3 WT, or FLAG-tagged Bub3 S135A cells are shown. Quantitative data in (*D*) were collected from 100 cells for each group. *E*, HeLa cells expressing vector, WT, or S135A Bub3 were stained with α-tubulin and DAPI to visualize microtubules (*green*) and chromosomes (*blue*), respectively. Data in (*F*) were collected from 160 mitotic cells for each group. Statistical analyses were conducted using *t* test; *p* values are presented. Bub3, benzimidazoles 3; DAPI, 4′,6-diamidino-2-phenylindole.

We further assessed chromosomal stability in cells expressing the vector, WT, or S135A mutant Bub3. We found that the expression of Bub3 S135A in HCT116 cells significantly increased the number of aneuploid cells compared with that in WT cells (Fig. 3, *A* and *B*). To detect the impact of the S/A mutation on mitotic division, we transfected the vector, FLAG-tagged WT, or S135A Bub3 plasmid into HeLa cells and found that the expression of Bub3 S135A resulted in a significantly higher percentage of cells with multiple nuclei, suggesting that Bub3 Ser135 phosphorylation is essential for mitotic cell division (Fig. 3, *C* and *D*). In addition, a remarkably higher percentage of cells with multipolar spindles was found in Bub3 S135A cells (Fig. 3, *E* and *F*). Together, our data indicate that mitotic phosphorylation of Bub3 Ser135 by ATM is required for the maintenance of chromosomal stability.

### Complementation of Bub3 S135 fails to rescue the SAC defect in Bub3 knockdown cells

**Figure 4:**
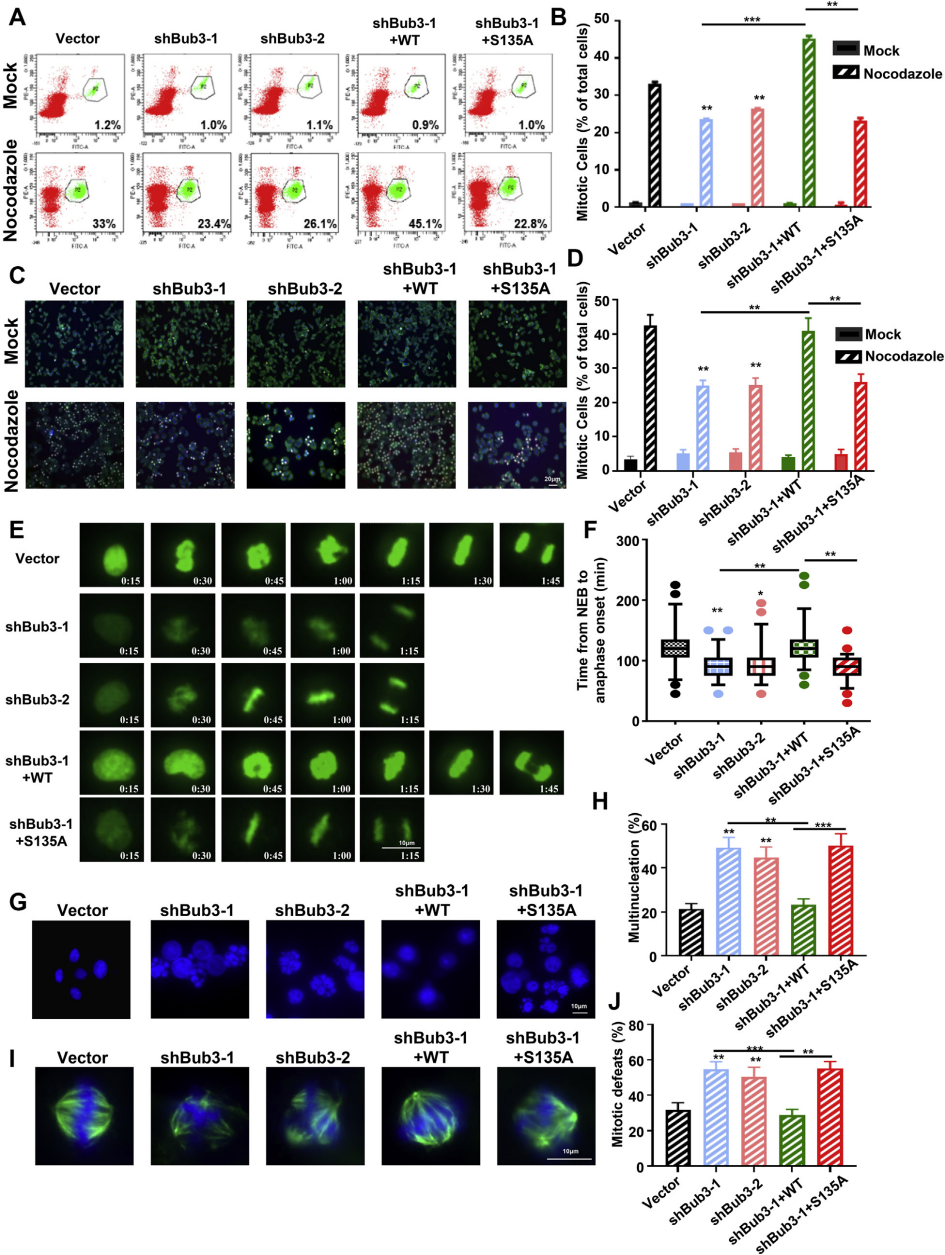
**Complementation of Bub3 S135 failed to rescue the SAC defect in Bub3 knockdown cells.** HeLa cells with stably knocking down of Bub3 were reconstituted with vector only, WT, or S135A mutant Bub3. They were used in the following experiments: *A* and *B*, cell cycle analysis and quantification. The isogenic cells were treated with mock or nocodazole before they were stained with a flow cytometry–based antiphosphor-histone-H3-Ser10 antibody to determine the mitotic index. *C*, cells were stained with α-tubulin and DAPI to visualize microtubules (*green*) and chromosomes (*blue*), respectively. *D*, data were collected from 100 cells for each group. *E*, the isogenic cells were transfected with H2B-GFP before they were imaged at the onset of mitosis to monitor chromosomal dynamics. Representative fluorescence images are shown. *F*, the average time from nuclear envelope breakdown (NEB) to anaphase onset in the cells was measured by time-lapse microscopy. *G*, multinucleated cells in the isogenic cells. Representative photos are shown. *H*, data in (*G*) were collected from 100 cells for each group. *I*, cells were stained with α-tubulin and DAPI to visualize microtubules (*green*) and chromosomes (*blue*), respectively. Data in (*J*) were collected from 160 mitotic cells for each group. Statistical analyses were conducted using *t* test; *p* values are presented. Bub3, benzimidazoles 3; DAPI, 4′,6-diamidino-2-phenylindole; SAC, spindle assembly checkpoint.

Because overexpressing a phosphorylation mutant protein might have pleotropic effects on the cellular phenotypes, we designed a set of complementation experiments by stably knocking down Bub3 in HeLa cells and reconstitution of the cells with WT or S135A mutant Bub3 (Fig. S3, *C* and *D*). Using flow cytometry (Fig. 4, *A* and *B*), IF (Fig. 4, *C* and *D*), and time-lapse microscopy (Fig. 4, *E* and *F*), we found that Bub3 knockdown cells displayed a significant reduction in mitotic arrest and chromosomal stability (Fig. 4, *A*–*J*). While WT Bub3 could rescue these phenotypes, S135A Bub3 failed to do so. Together, these data support the conclusion that Bub3 Ser135 phosphorylation is critical for the SAC and chromosomal stability.

### Bub3 Ser135 phosphorylation is required for the metaphase–anaphase transition in mitosis

**Figure 5:**
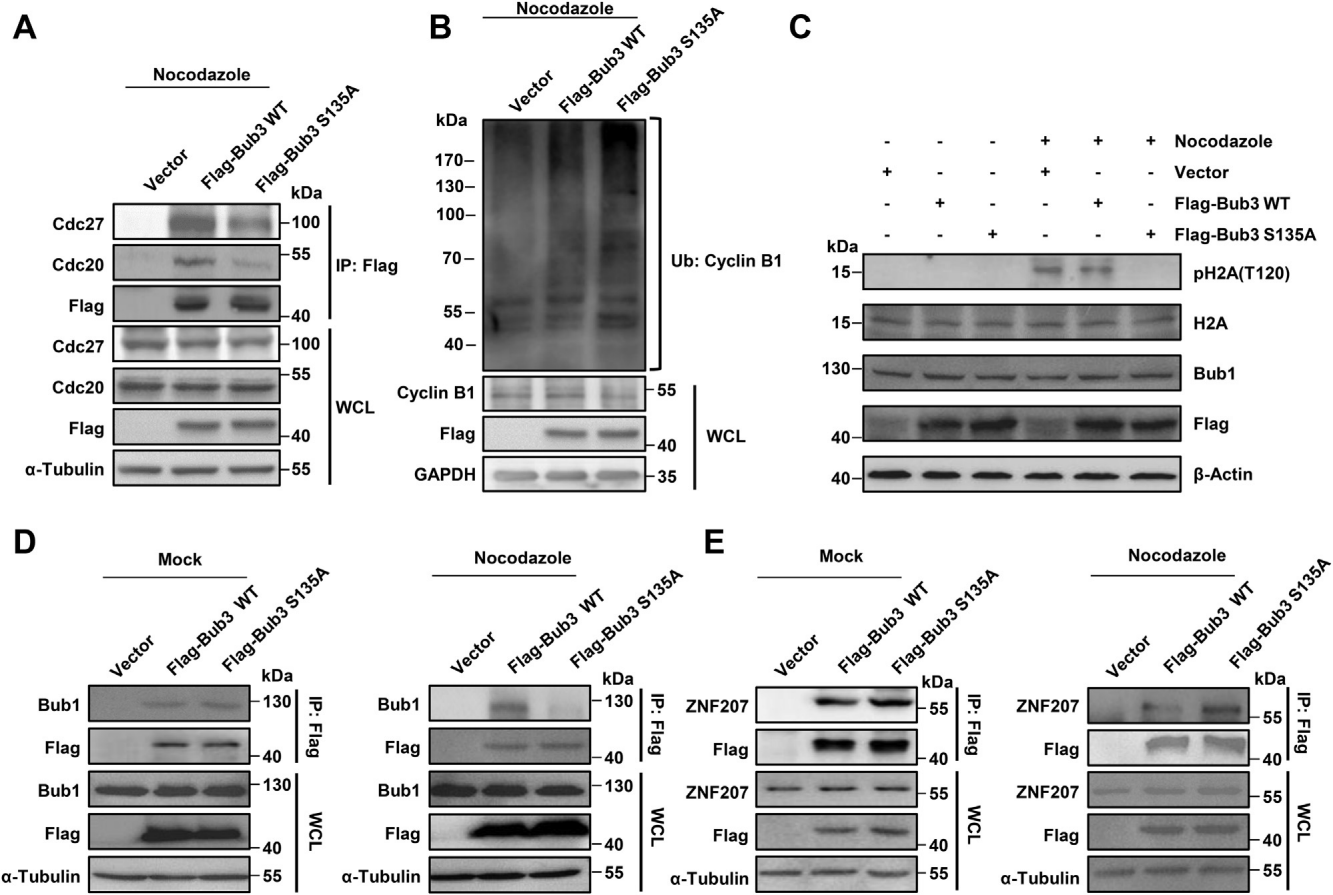
**Bub3 Ser135 phosphorylation is required for the metaphase–anaphase transition in mitosis.** HeLa cells transfected with vector, FLAG-Bub3 WT, or FLAG-Bub3 S135A were treated with nocodazole. *A*, immunoprecipitation of FLAG followed by Western blot with the anti-Cdc20 and anti-Cdc27 antibodies. *B*, immunoprecipitation of cyclin B1 followed by Western blot with the anti-Ub antibody. *C*, expression of pH2A (Thr120), H2A, FLAG, Bub1, and β-actin detected by Western blots using indicated antibodies. *D* and *E*, immunoprecipitation of FLAG-tagged proteins followed by Western blot using indicated antibodies. Bub3, benzimidazoles 3; Ser135, serine 135.

The SAC prevents the metaphase–anaphase transition by inhibiting the ubiquitylation and destruction of cyclin B1 by Cdc20-activated APC/C. To investigate how Bub3 Ser135 phosphorylation affects the metaphase–anaphase transition, we first examined the interaction between Bub3 and Cdc20 or Cdc27 (a subunit of APC/C). As shown in Figure 5*A*, S135A-mutated Bub3 significantly decreased its interaction with Cdc20 or Cdc27 when cells were treated with nocodazole. We also checked the ubiquitination of cyclin B1, the substrate of APC/C, and found that S135A-mutated Bub3 significantly increased the ubiquitination of cyclin B1 (Fig. 5*B*). These data indicate that Bub3 Ser135 phosphorylation is required for metaphase–anaphase transition.

During activation of the SAC, Bub3 is associated with the mitotic kinase Bub1, which phosphorylates histone H2A at threonine 120 (H2A-Thr120) (19, 20). We hypothesized that ATM-mediated Bub3 Ser135 phosphorylation might affect the activity of Bub1. Therefore, we examined the phosphorylation of H2A-Thr120. As shown in Figure 5*C*, the expression of S135A significantly decreased the phosphorylation signal. These data indicate that Bub3 Ser135 phosphorylation contributes to the execution of the SAC by activation of Bub1.

To investigate how Bub3 Ser135 phosphorylation contributes to Bub1 activation, we examined the interaction between Bub3 and Bub1 in the presence or the absence of nocodazole in cells expressing Bub3 WT or S135A. In unsynchronized cells, we found that Bub3 interacted with Bub1 and that the interaction was not affected when S135A was expressed (Fig. 5*D*). However, upon treatment with nocodazole, the S135A-mutated Bub3 significantly decreased its interaction with Bub1. These data indicate that Bub3 Ser135 is critical for interaction with Bub1 during mitosis.

Since ZNF207 is a component of the spindle matrix that binds to Bub3 and plays a critical role in SAC signaling pathways (21), we investigated whether the interaction between Bub3 and ZNF207 was affected by phosphorylation. We did not observe a change in the interaction of Bub3 with ZNF207 when Ser135 was mutated in unperturbed cells (Fig. 5*E*). However, the Bub3 S135A mutation increased the interaction with ZNF207 after nocodazole treatment. Taken together, our data indicate that Bub3 Ser135 phosphorylation increases Bub1 activity by promoting Bub3–Bub1 binding and competitively inhibiting Bub3–ZNF207 binding during mitosis.

In addition, we performed gene set enrichment analysis based on RNA-Seq to identify the signaling pathways associated with Bub3 Ser135 phosphorylation. Notably, we found that cell cycle pathways were significantly inhibited in the group of Bub3 S135A mutation compared with WT group (Fig. S4), suggesting that Bub3 Ser135 phosphorylation was closely related to cell cycle regulation.

### Bub3 Ser135 phosphorylation is also induced by IR

**Figure 6:**
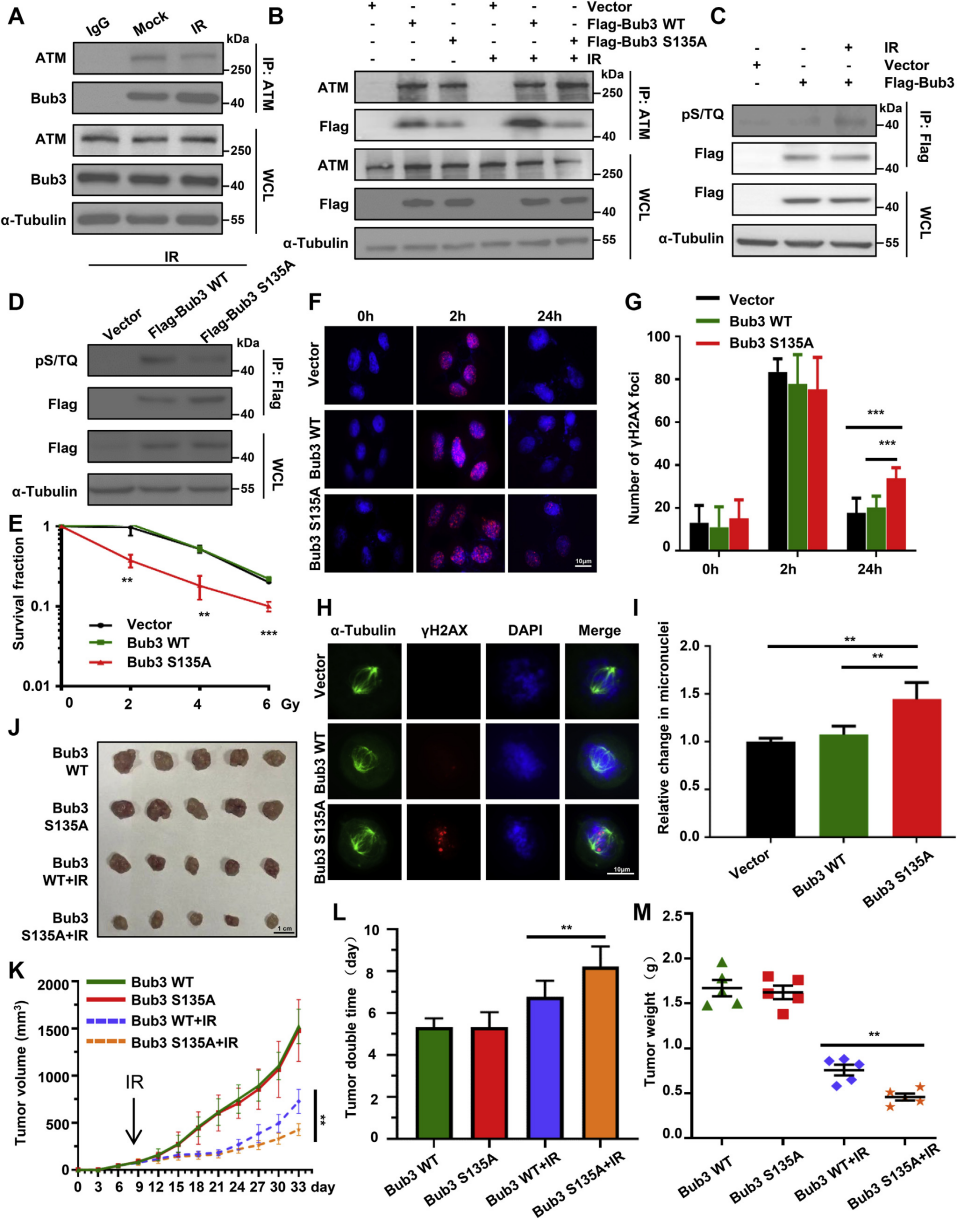
**Bub3 Ser135 phosphorylation is also induced by IR.***A*, HeLa cells irradiated with 0 Gy (Mock) or 6 Gy (IR) were immunoprecipitated with an anti-ATM antibody and analyzed by Western blot using indicated antibodies. *B*, HeLa cells transiently transfected with vector, FLAG-Bub3 WT, or FLAG-Bub3 S135A were irradiated with 0 Gy (Mock) or 6 Gy (IR) were immunoprecipitated with an anti-ATM antibody and analyzed by Western blot using indicated antibodies. *C*, immunoprecipitation of FLAG-tagged proteins followed by Western blot with the antiphosphor-S/TQ antibody. Cells transfected with the vector and FLAG-Bub3 WT were treated with mock or IR. *D*, HeLa cells transfected with the vector, FLAG-Bub3 WT, or FLAG-Bub3 S135A were treated with IR, and immunoprecipitation of FLAG-tagged proteins were immunoblotted with indicated antibodies. *E*, radiosensitivity was measured by the colony formation assay and shown are the survival fractions of HeLa cells stably expressing vector only, WT, or S135A Bub3. *F*, HeLa cells expressing vector only, WT, or S135A Bub3 were mock treated or IR treated. At indicated time points, they were fixed, permeabilized, and stained for γ-H2AX. Cell nuclei were stained with DAPI. *G*, the number of γ-H2AX foci per cell with the indicated time points was quantified using the ImageJ software. *H*, after IR, vector, Bub3 WT, and Bub3 S135A cells were treated with nocodazole for 17 h. Thereafter, the cells were fixed and subjected to immune fluorescence staining with anti-α-tubulin, γH2AX antibodies, and DAPI. *I*, micronuclei quantification of the vector, FLAG-Bub3 WT, or FLAG-Bub3 S135A cells, assessed by cell sorting. Shown are relative changes in micronuclei, and statistical analyses were conducted using *t* test; *p* values are presented. *J*, xenografts in nude mice were performed using HeLa cells stably expressing WT or S135A Bub3. Tumors were irradiated with a single dose of 10 Gy. Shown are tumors grown in mice when the experiments were finished. *K*, tumor growth curves after IR in xenograft mice are shown. Tumor volumes were measured every 3 days. *L*, relative growth delay of tumors. *M*, the weight of the tumors. γ-H2AX, gamma-H2A histone family member X; ATM, ataxia–telangiectasia mutated; Bub3, benzimidazoles 3; DAPI, 4′,6-diamidino-2-phenylindole; IR, ionizing radiation; Ser135, serine 135.

Because of the role of ATM and Bub3 in the DDR, we also tested whether Bub3 Ser135 phosphorylation could be induced by IR. We first investigated whether IR could induce the endogenous interaction of ATM and Bub3. As shown in Figure 6*A*, IR could increase the interaction between Bub3 and ATM. We also investigated that the interaction could be affected by the S135A mutation. As shown in Figure 6*B*, compared with Bub3 WT, S135A abrogated IR-induced interaction between Bub3 and ATM. We also pulled down FLAG-tagged Bub3 and examined it for S/TQ phosphorylation. We found that IR induced Bub3 S/TQ phosphorylation (Fig. 6*C*) and that the S135A mutation significantly reduced this phosphorylation (Fig. 6*D*). These data indicate that Bub3 Ser135 phosphorylation is also induced by IR.

### IR-induced Bub3 Ser135 phosphorylation is required for the DNA DSB repair

We further explored whether IR-induced Bub3 Ser135 phosphorylation is directly involved in the DDR. We found that both colony formation and 3-(4,5-dimethylthiazol-2-yl)-2,5-diphenyltetrazolium bromide assays showed hypersensitivity to IR in cells expressing S135A (Figs. 6*E* and S5, *A* and *B*). To further study defects in the DSB repair when Bub3 Ser315 is mutated, we examined the accumulation of γ-H2AX, one of the earliest events in the DDR (22). We found that a prolonged presence of γ-H2AX foci was observed in cells expressing Bub3 S135A (Fig. 6, *F* and *G*), indicating that the efficiency of the DNA repair decreased when Bub3 S135A was expressed. Further supporting this conclusion is the evidence showing the accumulation of γ-H2AX foci in mitotic cells expressing Bub3 S135A (Fig. 6*H*).

To assess whether the defected DNA repair caused by the Bub3 S135A mutation had an impact on genomic instability, we measured micronuclei formation in response to IR as micronuclei formation is an indicator of genomic instability (23). We found that in response to IR, the number of cells containing micronuclei significantly increased in S135A-expressing cells, compared with that in the vector or Bub3 WT cells (Fig. 6*I*), indicating that Bub3 S135A is associated with enhanced genomic instability.

To further support this conclusion, we conducted a xenograft experiment in nude mice (Fig. 6*J*). We found that tumors in the Bub3 S135A mutated group were much more sensitive to IR than those in the WT group (Fig. 6, *K*–*M*). These results indicate that Bub3 Ser135 phosphorylation promotes radiosensitivity *in vivo*.

### Complementation of Bub3 S135 fails to rescue radiosensitivity and nonhomologous end-joining repair defects in Bub3 knockdown cells

**Figure 7:**
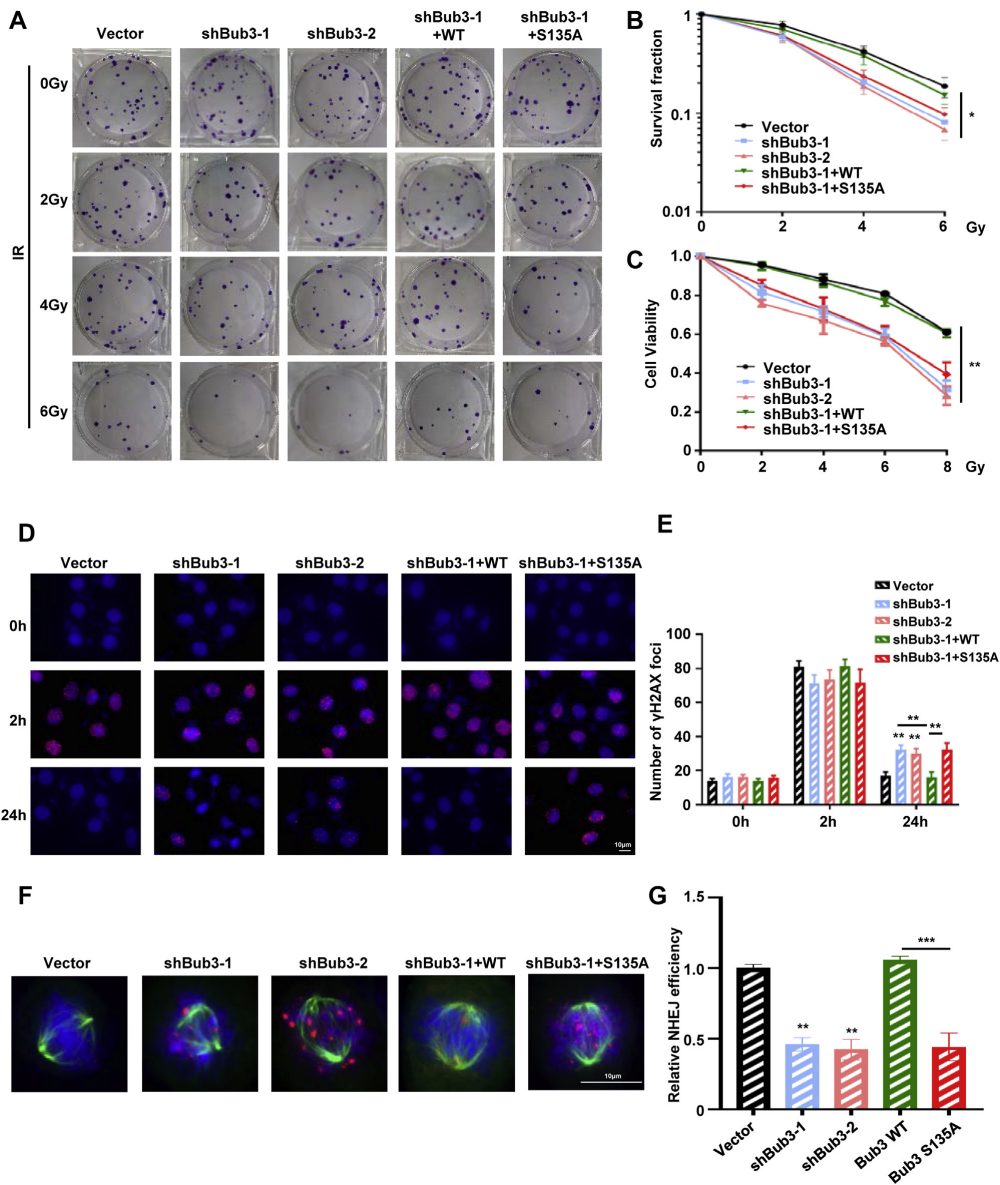
**Complementation of Bub3 S135 failed to rescue radiosensitivity and NHEJ repair defect in Bub3 knockdown cells.***A*, the colony formation assay was performed on vector, shBub3-1, shBub3-2, shBub3-1 + WT, or shBub3 + S135A cells 14 days after exposure to IR. Shown are representative colonies. *B*, the number of clones was counted under the microscope. The survival fraction is shown. *C*, radiosensitivity is measured by the MTT assay, and cell viability as a function of IR doses is shown. *D*, IR-induced γ-H2AX focus formation was measured in the isogenic cells by immunofluorescence microscopy. Cell nuclei were stained with DAPI (*blue*). Shown are representative images. *E*, the number of γ-H2AX foci per cell at the indicated time points was quantified using the ImageJ software. *F*, after IR, vector, shBub3-1, shBub3-2, shBub3-1 + WT, and shBub3 + S135A cells were treated with nocodazole for 17 h. Thereafter, the cells were fixed and subjected to immune fluorescence staining with anti-α-tubulin (*green*), γH2AX antibodies (*red*), and DAPI (*blue*). *G*, the NHEJ efficiency was analyzed by an NHEJ reporter system in U2OS cells transient transfected with vector, shBub3-1, shBub3-2, shBub3-1 + WT, or shBub3 + S135A. γ-H2AX, gamma-H2A histone family member X; Bub3, benzimidazoles 3; DAPI, 4′,6-diamidino-2-phenylindole; IR, ionizing radiation; MTT, 3-(4,5-dimethylthiazol-2-yl)-2,5-diphenyltetrazolium bromide; NHEJ, nonhomologous end-joining.

Using the isogenic cell lines with complementation of WT or S135A in Bub3 knockdown cells, we found that Bub3 S135A complementation showed similar hypersensitivity to IR compared with Bub3 knockdown cells (Fig. 7, *A*–*C*). Bub3 S135A complementation also showed prolonged existence of γ-H2AX as well as enhanced micronuclei formation (Fig. 7, *D* and *E*). Furthermore, Bub3 S135A complemented cells showed γ-H2AX in mitotic cells in response to IR, indicating an impaired DNA repair process even when cells entering mitosis (Fig. 7*F*). Nonhomologous end-joining (NHEJ) efficiency was then analyzed by the NHEJ reporter assay in U2OS cells. We found that cells transfected with Bub3 shRNA showed a significantly impaired NHEJ efficiency (Fig. 7*G*), supporting the notion that Bub3 is involved in DNA repair. Meanwhile, compared with Bub3 WT, cells expressing S135A showed a lowered NHEJ efficiency, indicating that Bub3 Ser135 phosphorylation participates in NHEJ.

### Downstream proteins of the ATM–Bub3 pathway in response to IR are independent to those in mitosis

**Figure 8:**
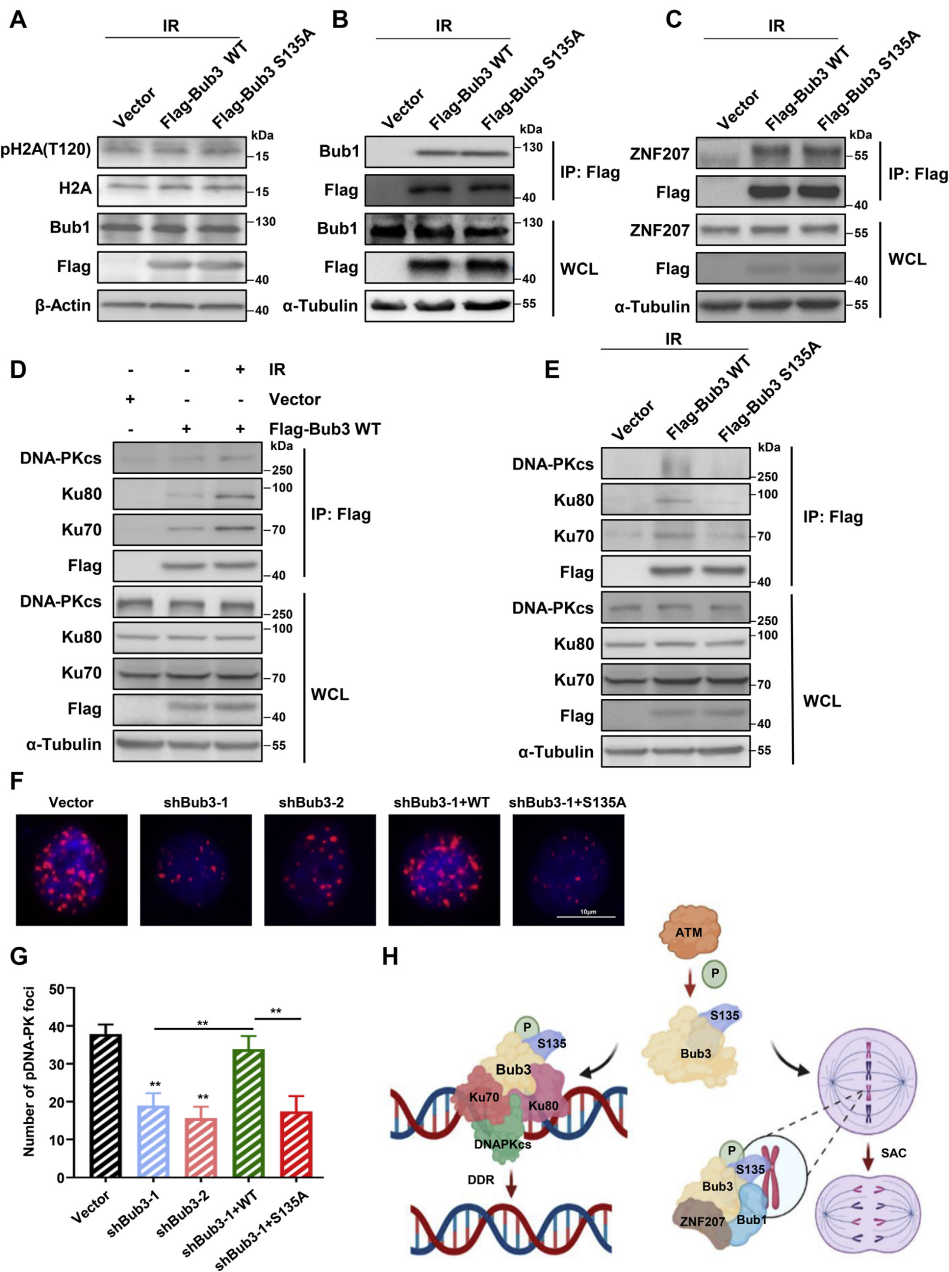
**S135A abrogates Bub3 interaction with Ku70–Ku80–DNA-PKcs and DNA-PKcs focus formation in response to IR.***A*, expression of pH2A (Thr120), H2A, FLAG, Bub1, and β-actin in cells transfected with vector, FLAG-Bub3 WT, or FLAG-Bub3 S135A constructs, treated with IR, and assessed by Western blot. *B* and *C*, immunoprecipitation of FLAG-tagged proteins followed by Western blot with the anti-Bub1 (*B*) or ZNF207 (*C*) antibody. *D*, immunoprecipitation of FLAG-tagged proteins followed by Western blot with the anti-Ku70, anti-Ku80, anti-DNA-PKcs, or anti-FLAG antibody. *E*, immunoprecipitation of FLAG-tagged proteins followed by Western blot with the anti-Ku70, anti-Ku80, anti-DNA-PKcs, or anti-FLAG antibody. *F*, immunofluorescence microscopy was conducted in the isogenic HeLa cells 2 h following IR. Cells were stained for pDNA-PKcs (*red*), and the nuclei were stained with DAPI (*blue*). *G*, the number of pDNA-PKcs foci per cell was quantified using ImageJ software. *H*, the schematic model for ATM-mediated Bub3 Ser135 phosphorylation in the SAC and DNA DSB repair. ATM, ataxia–telangiectasia mutated; Bub3, benzimidazoles 3; DAPI, 4′,6-diamidino-2-phenylindole; DSB, DNA double-stranded break; IR, ionizing radiation; SAC, spindle assembly checkpoint.

We tested whether the ATM–Bub3 pathway affected downstream proteins similar to those in mitosis. However, we found no noticeable changes in phosphorylation of H2A-Thr120 (Fig. 8*A*) in response to IR. Since Bub3 Ser135 phosphorylation increases Bub1 activity by promoting Bub3–Bub1 binding and competitively inhibiting Bub3–ZNF207 binding during mitosis, we further explored whether these interactions were disturbed in response to IR. Interestingly, we found that the expression of Ser135A had no effect on the interactions of Bub3–Bub1 or Bub3–ZNF207 in response to IR (Fig. 8, *B* and *C*). These observations indicate that IR-induced ATM phosphorylation of Bub3 Ser135 has an independent downstream pathway compared with that in mitosis.

### S135A abrogates Bub3 interaction with Ku70–Ku80–DNA-PKcs and DNA-PKcs focus formation in response to IR

To gain insights into the functional significance of Bub3 Ser135 phosphorylation in NHEJ repair, we aimed to identify a potential ATM/Bub3-related complex induced by IR. Since the complex of Ku70, Ku80, and DNA-PKcs plays a crucial role in the NHEJ pathway (24, 25), we examined the possible interactions between Bub3 and these proteins in response to IR. Ku70, Ku80, and DNA-PKcs showed a significant increase after IR in their interactions with FLAG-tagged Bub3 (Fig. 8*D*). However, the Bub3 S135A mutation showed a significant reduction in the interaction (Fig. 8*E*), indicating that the Ser135 residue is essential for the Ku70–Ku80–DNA-PKcs/Bub3 interaction in DDR. Furthermore, we also enumerated IR-induced *p*DNA-PKcs focus formation (Fig. 8, *F* and *G*). Similarly, complementation of Bub3 S135A failed to rescue the defect in IR-induced *p*DNA-PKcs focus formation in Bub3 knockdown cells, indicating that Bub3 Ser135 phosphorylation is critical for activation of DNA-PKcs in response to DSBs. In addition, to investigate whether these interactions participate in mitotic signaling, we also examined the Bub3–Ku70–Ku80 complex during mitosis. As shown in Fig. S6, we found that the expression of S135A had no effect on the interaction of Bub3–Ku70–Ku80 when cells were treated with nocodazole.

## Discussion

Activation of signaling transduction in the face of cellular dynamic changes often depends on elaboration of post-translational modifications (26, 27). Kinase-mediated protein phosphorylation plays an essential role in regulating protein function and subsequent phenotypes (28). ATM, as a pivotal protein kinase in the DDR, activates various downstream molecules by phosphorylation in response to DNA DSBs (29). Proteomics studies have identified over 700 proteins that are substrates of ATM-mediated phosphorylation (12). Extensive efforts have been made to study ATM-mediated pathways during interphase checkpoints upon DNA damage. However, the whole picture of the role of ATM in mitosis remains unclear. A critical step that can advance the study has been achieved in this study by identifying proteins that are putative substrates of ATM in mitosis by a quantitative phosphorproteomics assay. Not surprisingly, these proteins are heavily involved in cell cycle regulation and DDR, supporting the importance of crosstalk interaction between the DDR and mitotic control.

Our results demonstrate that Bub3 is a *bona fide* ATM substrate and that phosphorylation represents dual-functional regulation of both the SAC and DNA DSB repair. As an SAC protein, Bub3 is responsible for ensuring accurate chromosome segregation mainly by forming the mitotic checkpoint complex that blocks and stabilizes APC/C, thereby preventing an advance to anaphase onset (30). Our findings further the understanding that the functional significance of Bub3 Ser135 phosphorylation is required for the metaphase–anaphase transition and the promotion of Bub1 activity during mitosis. In addition, our data show that cells with S135A mutation have significantly increased aneuploid cells, indicating that Bub3 Ser135 phosphorylation is required for the maintenance of the mitotic spindle checkpoint.

Growing evidence has shown that the DDR pathway and mitotic checkpoint crosstalk. In our study, we demonstrate that ATM phosphorylates Bub3 Ser135 in response to IR and mitosis (Fig. 8*H*). However, unlike in mitosis, ATM phosphorylation of Bub3 Ser135 does not affect Bub1 activity and the binding of Bub3–Bub1 in response to IR, but it affects the KU70–Ku80–DNA-PKcs interaction. However, these interactions do not participate in the mitotic response. These results demonstrate that ATM-mediated Bub3 Ser135 phosphorylation has independent downstream pathways in DSB repair and mitosis. In the interaction between DSB repair and SAC, ATM-mediated Bub3 phosphorylation appears to be at the crossroad where the two pathways part for further functions.

It is noted that there might be feedback loop between ATM–Bub3 interaction and phosphorylation. This is supported by the fact that the phosphorylation site mutation reduced interaction (Fig. 1, *E* and *F*). Meanwhile, it is likely that ATM-mediated phosphorylation further strengthens the interaction through alteration of the secondary structure of Bub3.

As shown in our results (Fig. S6), the interaction between Bub3 and Ku70 in mitotic cells is not dependent on the phosphorylation status, as the S135A mutant Bub3 can still be immunoprecipitated with Ku70. However, the interaction between WT Bub3 and Ku70 is enhanced when cells (unsynchronized) undergo IR-induced DNA damage (Fig. 8*D*), whereas the interaction between the S135A mutant and Ku70 reduced compared with WT (Fig. 8*E*). On the contrary, the Bub3 and Bub1 interaction was not affected by IR but by mitotic stress (Fig. 8*B*). Therefore, these changes are indeed dependent on specific situations (*i.e.*, mitotic dependent or DNA damage dependent).

In the clinical setting, Bub3 is overexpressed in multiple cancers, and overexpression of Bub3 is associated with poor survival in patients with adrenocortical carcinoma and oral cancer (31, 32). Enhanced Bub3 expression may lead to Bub3 hyperphosphorylation, which could enhance the execution of the SAC and DSB repair, resulting in resistance to treatment. In addition, germline mutation of Bub3 is proposed to be a risk factor for colorectal cancer and pancreatic adenocarcinoma (33, 34). These mutations may affect the phosphorylation of Bub3. Given that the inhibition of SAC protein activities sensitizes cancer cells to chemotherapy and radiotherapy (35, 36, 37), our findings on the dual function of ATM phosphorylation make it a plausible target for radiosensitization and chemosensitization.

In conclusion, our data demonstrate that Bub3 Ser135 phosphorylation mediated by ATM is essential in the SAC and DSB repair, whereas mutated Bub3 Ser135 significantly reduces the capability of SAC and NHEJ repair, underpinning the intertwined roles of ATM in preventing genomic instability.

## Experimental procedures

### Cells and cell culture

The human cervical cancer cell line, HeLa, the colorectal cancer cell line, HCT116, and the human bone osteosarcoma epithelial cell line, U2OS, were purchased from the American Type Culture Collection. HCT116 and U2OS cells were routinely cultured in RPMI1640 medium (Invitrogen), and HeLa cells were cultured in Dulbecco's modified Eagle's medium (Invitrogen) supplemented with 10% fetal bovine serum (Gibco), 100 U/ml penicillin (Gibco), and 100 μg/ml streptomycin (Gibco). Cells were incubated in a humidified atmosphere containing 5% CO_2_ at 37 °C and confirmed to be mycoplasma negative. To generate stable cell lines, the pBabe-puro–based FLAG-Bub3-WT, FLAG-Bub3-S135A, and hemagglutinin (HA)-ATM constructs were transfected into 293T cells for retroviral packaging and subsequent transduction. Stable cells were selected with puromycin (2 μg/ml) (Gibco). To obtain cells in mitosis, cells were treated with 200 nM nocodazole (Selleck) for 17 h.

### Plasmid construction, lentivirus production, and transfection

The target sequences of Bub3 for constructing lentiviral shRNAs in the pLKO.1-Hygro vector are 5′-GCAATAGCGTCATCATATAT-3′ (shBub3-1) and 5′-GGGTTATGTATTAAGCTCTATT-3′ (shBub3-2). For rescue experiments, the Bub3-WT or Bub3-S135A plasmid (mutations underlined: GCGATCGCATCCTCCTACAT) resistant to the shBub3-1 (5′-GCAATAGCGTCATCATATAT-3′) was cloned into a FLAG-tagged pBabe-puro lentivector. To generate stable cell lines, plasmids were transfected into 293T cells for retroviral packaging and subsequent transduction. Stably expressed clones were selected by RT–quantitative PCR (RT–qPCR) and immunoblotting assays after hygromycin B (500 μg/ml) or puromycin (2 μg/ml) (Gibco) treatment.

### RT–qPCR

Total RNAs were extracted using Trizol reagent (Invitrogen). For quantitative analysis of Bub3 expression, complementary DNA synthesis was carried out using 1 μg of total RNA and the iScript cDNA Synthesis Kit (Bio-Rad) according to manufacturer's instructions. RT–qPCR was performed using iQ SYBR Green supermix (Bio-Rad) using the CFX96 real-time PCR Detection System (Bio-Rad). Relative gene expression levels of mRNAs were normalized using the reference gene GAPDH. The Bub3 forward primer sequence was 5′-GGTTCTAACGAGTTCAAGCTGA-3′, and the reverse primer sequence was 5′-GGCACATCGTAGAGACGCAC-3′. The GAPDH forward primer sequence was 5′-GGAGCGAGATCCCTCCAAAAT-3′, and the reverse primer sequence was 5′-GGCTGTTGTCATACTTCTCATGG-3′. The threshold cycle (Ct) values were analyzed using the comparative Ct (−ΔCt) method.

### SILAC and protein digestion

HA-tagged ATM was stably expressed in HeLa cells. Cells were grown for five to six generations and treated with 200 nM nocodazole in “heavy” medium. Cells grown in “light” medium were treated with dimethyl sulfoxide. About 17 h following nocodazole treatment, cell lysates were harvested and mixed and then pulled down with the HA antibody (catalog no.: 3724; Cell Signaling Technology). The interacting proteins were eluted with elution buffer, and protein digestion was performed as previously described (38).

### Phosphopeptide enrichment and LC–MS/MS

After digestion, the peptides were quantified using a quantitative colorimetric peptide assay (catalog no.: 23275; Thermo Fisher Scientific). Per milligram of peptides were used for phosphopeptide enrichment with a TiO_2_ phosphopeptide enrichment kit following the manufacturer's instructions (catalog no.: A32993; Thermo Fisher Scientific). TiO_2_-enriched phosphopeptides were analyzed by LC–MS/MS.

### Western blot and immunoprecipitation

Total protein was extracted from cells using radioimmunoprecipitation assay and quantified using the bicinchoninic acid method. Protein samples were separated on a 10% SDS polyacrylamide gel and transferred onto polyvinylidene fluoride membranes. After blocking with 5% milk–Tris-buffered saline with Tween-20 for 1 h, the proteins were incubated with a primary antibody at 4 °C overnight and a horseradish peroxidase–conjugated secondary antibody (catalog no.: 7076 for mouse, catalog no.: 7074 for rabbit; Cell Signaling Technology). The membranes were visualized using the ImageJ software (National Institutes of Health). For immunoprecipitation, cells were lysed for 30 min at 4 °C, and supernatants were collected. Cell lysates were incubated with an antibody at 4 °C overnight, and agarose beads were added and incubated for 2 h at 4 °C. The beads were washed three times with lysis buffer, boiled with 5× SDS loading buffer for 5 min, and then analyzed by Western blot using specific antibodies.

### *In vitro* binding assay

Binding assays were conducted using biolayer interferometry on the Octet RED (Pall ForteBio) with protein A-labeled dip-and-read biosensors. Recombinant and constitutively active ATM was loaded onto the biosensors at a concentration of 2 ng/μl. Loading was performed for 300 s, followed by a baseline reading, an association reading for 300 s, and a dissociation reading for 600 s. Concentration of Bub3 and ZNF207 peptides ranged from 0.74 to 60 μM in 3:1 serial dilution for initial binding experiments. The binding data were analyzed using the Octet software analysis system.

### *In vitro* kinase assay

The *in vitro* kinase assay was performed according to the protocol described by Millipore. Briefly, 10 ng of purified and constitutively active ATM was incubated with 6 μM of Bub3 WT and S135A Bub3 peptides comprising 10 amino acids in the presence of the reaction buffer. An ATP solution (250 μM) containing magnesium and manganese acetate was added to initiate the reaction. The reaction was allowed to proceed for 1 h at room temperature and stopped using the ADP Glo reagent (Promega). Absorbance was measured using a Synergy 4 plate reader (BioTek).

### Flow cytometry

To test mitotic arrest, cells were seeded into dishes of 6 cm, incubated at 37 °C in 5% CO_2_ for 24 h, and then harvested and fixed in 70% ethanol. After permeabilization with 0.1% Triton X-100 (Sigma) on ice for 10 min and blocking in PBS containing 1% bovine serum albumin for 30 min, the fixed cells were incubated with antiphosphor-histone-H3-Ser10 antibody (catalog no.: 53348; Cell Signaling Technology) for 2 h at room temperature and fluorescein isothiocyanate–conjugated secondary antibody for 25 min in the dark. The cells were then stained with 25 μg/ml propidium iodide (Solarbio), and the results were analyzed using an Fluorescence-activated cell sorting Calibur flow cytometer (Becton, Dickinson and Company) with CellQuest software (Becton, Dickinson and Company).

### Live cell time-lapse imaging

To monitor chromosome dynamics, HeLa cells were transfected with GFP-tagged H2B plasmid (AddGene). After 48 h, the cells were monitored in the chamber of a Lionheart FX automated microscope (BioTek). Images were acquired every 15 min for 24 h and analyzed with Gen5 3.0 (BioTek).

### Chromosome spread and karyotype analysis

Cells in dishes of 10 cm were accumulated in mitosis by treatment with 0.05 μg/ml colcemid for 3 h. All cells were collected by centrifugation and resuspended in 0.075 M KCl for 10 min at room temperature. Then, the cells were centrifuged and fixed with an ice-cold fixative agent, consisting of methanol and acetic acid in a 3:1 ratio. Cells were rewashed two times with a fresh fixative agent resuspension. Three drops of cells were dropped onto precleaned microscope slides and stained with Giemsa stain solution (Solarbio). At least 50 metaphase spreads were counted for each transfected cell line.

### pEGFP-Pem1-Ad2 (NHEJ) reporter assay

The NHEJ assay was performed in U2OS cells with stably transfected pEGFP-Pem1-Ad2 plasmid. The vector, shBub3-1, shBub3-2, shBub3-1 + WT, or shBub3 + S135A, plasmid was cotransfected with the I-SecI plasmid in the stable cell line. After 48 h, cells were harvested and analyzed using fluorescence-activated cell sorting to determine the percent of GFP-positive cells.

### RNA-Seq analyses

HeLa cells were plated in 6 cm culture dishes at a density of 6 × 10^5^ cells/dish and incubated overnight at 37 °C in 5% CO_2_ for attachment. Exponentially growing ells were transfected with BUb3 WT or S135A plasmids for 48 h, respectively. RNA extraction, RNA quality determination, and the results of RNA-Seq analysis were conducted in Novogene, Inc.

### Irradiation

We used 6 MV X-ray irradiation (600CD; Varian) at a dose rate of 4 Gy/min.

### Micronuclei quantification

The micronuclei staining kit was purchased from IntelliCyt Corporation. Cells were plated in 384-well plates at a density of 3000 cells/well. The cells were then treated with radiation, and fresh medium was added 24 h after radiation. After 72 to 96 h of radiation, the cells were stained according to the manufacturer's protocol. Cell sorting was performed using IQue (IntelliCyt Corporation). The analysis was performed using the Forecyt software (IntelliCyt Corporation).

### 3-(4,5-Dimethylthiazol-2-yl)-2,5-diphenyltetrazolium bromide

Transfected cells were treated with or without IR. All cells were supplemented with fresh media 24 h after radiation exposure. About 72 h following the manufacturer's instructions (Acros Organics), absorbance was read at 570 nm using a Synergy 4 plate reader (BioTek).

### IF

Cells (4 × 10^4^) were seeded onto coverslips (Thermo Fisher Scientific) and incubated in 5% CO_2_ at 37 °C overnight. Then, the cells were washed twice with PBS, immediately followed by fixation with 4% paraformaldehyde for 1 h at room temperature. After permeabilization with 0.2% PBS with Tween-20 for 10 min and blocking with 5% bovine serum albumin in PBS with Tween-20 for 1 h, the cells were incubated with anti-γ-H2AX (catalog no.: 9718; Cell Signaling Technology), antiphosphor-histone-H3-Ser10 antibody (catalog no.: 53348; Cell Signaling Technology), and anti-α-tubulin antibody (catalog no.: 3873; Cell Signaling Technology) overnight at 4 °C. Cells were then incubated with secondary fluorochrome-labeled antibodies (Jackson ImmunoResearch Laboratories) for 1 h at 37 °C. The coverslips were mounted using Prolong Diamond Antifade reagent containing 4′,6-diamidino-2-phenylindole (Invitrogen). Cell images were acquired using an IF microscope (Ziess).

### Colony formation assay

A colony formation assay was performed to examine the effect of Bub3 Ser135 on the sensitivity of tumor cells to radiotherapy. Briefly, cells were seeded in 60 mm^2^ culture dishes at a suitable cell density (1 × 10^2^–8 × 10^2^ cells/well) overnight and then exposed to a series of doses (0, 2, 4, and 6 Gy) of IR, followed by incubation for additional 14 days. Colonies were fixed with 100% methanol for 15 min and then stained with 1% crystal violet for 20 min. The number of colonies with more than 50 cells was counted.

### Xenografts in nude mice

Four-week-old female BALB/c nu/nu mice (Beijing Experimental Animal Center) were used for *in vivo* studies. All animal studies were performed following the animal procedures approved by the Institutional Animal Care and Use Committee of Tianjin Medical University Cancer Institute and Hospital and are consistent with the national regulatory standards. HeLa cells (5 × 10^6^) expressing either Bub3 WT or Bub3 S135A were injected into the right thigh of nude mice. After 10 days, the xenografts were irradiated with 10 Gy (2 GyX5). Two-dimensional tumor sizes were measured every 3 days for 33 days.

### Statistical analysis

All statistical analyses were performed in at least three independent experiments. Student's *t* test or one-way ANOVA for two or multiple group comparisons was performed using SPSS 21 (IBM). Statistical significance was set at *p* ≤ 0.05.

## Data availability

All data that support the findings of this study are available from the corresponding authors upon reasonable request.

## Supporting information

This article contains supporting information.

## Conflict of interest

The authors declare that they have no conflicts of interest with the contents of this article.

## References

[1] Jeggo P., Pearl L., Carr A. (2016). DNA repair, genome stability and cancer: A historical perspective. Nat. Rev. Cancer.

[2] Pilié P., Tang C., Mills G., Yap T. (2019). State-of-the-art strategies for targeting the DNA damage response in cancer. Nat. Rev. Clin. Oncol..

[3] O'Connor M. (2015). Targeting the DNA damage response in cancer. Mol. Cell.

[4] Blackford A., Stucki M. (2020). How cells respond to DNA breaks in mitosis. Trends Biochem. Sci..

[5] Toulany M. (2019). Targeting DNA double-strand break repair pathways to improve radiotherapy response. Genes.

[6] Lee J., Paull T. (2005). ATM activation by DNA double-strand breaks through the Mre11-Rad50-Nbs1 complex. Science.

[7] Boohaker R., Xu B. (2014). The versatile functions of ATM kinase. Biomed. J..

[8] Khanna K., Lavin M., Jackson S., Mulhern T. (2001). ATM, a central controller of cellular responses to DNA damage. Cell Death Differ..

[9] Levy A., Lang A. (2018). Ataxia-telangiectasia: A review of movement disorders, clinical features, and genotype correlations. Mov. Disord..

[10] Lara-Gonzalez P., Westhorpe F., Taylor S. (2012). The spindle assembly checkpoint. Curr. Biol..

[11] Yang C., Tang X., Guo X., Niikura Y., Kitagawa K., Cui K., Wong S.T., Fu L., Xu B. (2011). Aurora-B mediated ATM serine 1403 phosphorylation is required for mitotic ATM activation and the spindle checkpoint. Mol. Cell.

[12] Matsuoka S., Ballif B.A., Smogorzewska A., McDonald E.R., Hurov K.E., Luo J., Bakalarski C.E., Zhao Z., Solimini N., Lerenthal Y., Shiloh Y., Gygi S.P., Elledge S.J. (2007). ATM and ATR substrate analysis reveals extensive protein networks responsive to DNA damage. Science.

[13] Yang C., Hao J., Kong D., Cui X., Zhang W., Wang H., Guo X., Ma S., Liu X., Pu P., Xu B. (2014). ATM-mediated Mad1 Serine 214 phosphorylation regulates Mad1 dimerization and the spindle assembly checkpoint. Carcinogenesis.

[14] Musacchio A. (2015). The molecular biology of spindle assembly checkpoint signaling dynamics. Curr. Biol..

[15] Eliezer Y., Argaman L., Kornowski M., Roniger M., Goldberg M. (2014). Interplay between the DNA damage proteins MDC1 and ATM in the regulation of the spindle assembly checkpoint. J. Biol. Chem..

[16] Landmann C., Pierre-Elies P., Goutte-Gattat D., Montembault E., Claverie M.C., Royou A. (2020). The Mre11-Rad50-Nbs1 complex mediates the robust recruitment of Polo to DNA lesions during mitosis in *Drosophila*. J. Cell Sci..

[17] Derive N., Landmann C., Montembault E., Claverie M.C., Pierre-Elies P., Goutte-Gattat D., Founounou N., McCusker D., Royou A. (2015). Bub3-BubR1-dependent sequestration of Cdc20Fizzy at DNA breaks facilitates the correct segregation of broken chromosomes. J. Cell Biol..

[18] Guleria A., Chandna S. (2016). ATM kinase: Much more than a DNA damage responsive protein. DNA Repair.

[19] Kawashima S., Yamagishi Y., Honda T., Ishiguro K., Watanabe Y. (2010). Phosphorylation of H2A by Bub1 prevents chromosomal instability through localizing shugoshin. Science.

[20] Yu H., Tang Z. (2005). Bub1 multitasking in mitosis. Cell Cycle.

[21] Jiang H., He X., Wang S., Jia J., Wan Y., Wang Y., Zeng R., Yates J., Zhu X., Zheng Y. (2014). A microtubule-associated zinc finger protein, BuGZ, regulates mitotic chromosome alignment by ensuring Bub3 stability and kinetochore targeting. Dev. Cell.

[22] Bonner W.M., Redon C.E., Dickey J.S., Nakamura A.J., Sedelnikova O.A., Solier S., Pommier Y. (2008). GammaH2AX and cancer. Nat. Rev. Cancer.

[23] Terradas M., Martín M., Genescà A. (2016). Impaired nuclear functions in micronuclei results in genome instability and chromothripsis. Arch. Toxicol..

[24] Falck J., Coates J., Jackson S. (2005). Conserved modes of recruitment of ATM, ATR and DNA-PKcs to sites of DNA damage. Nature.

[25] Tomimatsu N., Tahimic C.G., Otsuki A., Burma S., Fukuhara A., Sato K., Shiota G., Oshimura M., Chen D.J., Kurimasa A. (2007). Ku70/80 modulates ATM and ATR signaling pathways in response to DNA double strand breaks. J. Biol. Chem..

[26] Rigbolt K., Blagoev B. (2012). Quantitative phosphoproteomics to characterize signaling networks. Semin. Cell Dev. Biol..

[27] Olsen J.V., Blagoev B., Gnad F., Macek B., Kumar C., Mortensen P., Mann M. (2006). Global, *in vivo*, and site-specific phosphorylation dynamics in signaling networks. Cell.

[28] Tarrant M., Cole P. (2009). The chemical biology of protein phosphorylation. Annu. Rev. Biochem..

[29] Paull T. (2015). Mechanisms of ATM activation. Annu. Rev. Biochem..

[30] Alfieri C., Chang L., Zhang Z., Yang J., Maslen S., Skehel M., Barford D. (2016). Molecular basis of APC/C regulation by the spindle assembly checkpoint. Nature.

[31] Subramanian C., Cohen M. (2019). Over expression of DNA damage and cell cycle dependent proteins are associated with poor survival in patients with adrenocortical carcinoma. Surgery.

[32] Silva P.M.A., Delgado M.L., Ribeiro N., Florindo C., Tavares Á.A., Ribeiro D., Lopes C., do Amaral B., Bousbaa H., Monteiro L.S. (2019). Spindly and Bub3 expression in oral cancer: Prognostic and therapeutic implications. Oral Dis..

[33] Shindo K., Yu J., Suenaga M., Fesharakizadeh S., Cho C., Macgregor-Das A., Siddiqui A., Witmer P.D., Tamura K., Song T.J., Navarro Almario J.A., Brant A., Borges M., Ford M., Barkley T. (2017). Deleterious germline mutations in patients with apparently sporadic pancreatic adenocarcinoma. J. Clin. Oncol..

[34] de Voer R.M., Geurts van Kessel A., Weren R.D., Ligtenberg M.J., Smeets D., Fu L., Vreede L., Kamping E.J., Verwiel E.T., Hahn M.M., Ariaans M., S.pruijt L., van Essen T., Houge G., Schackert H.K. (2013). Germline mutations in the spindle assembly checkpoint genes BUB1 and BUB3 are risk factors for colorectal cancer. Gastroenterology.

[35] Morales A.G., Pezuk J.A., Brassesco M.S., de Oliveira J.C., de Paula Queiroz R.G., Machado H.R., Carlotti C.G., Neder L., de Oliveira H.F., Scrideli C.A., Tone L.G. (2013). BUB1 and BUBR1 inhibition decreases proliferation and colony formation, and enhances radiation sensitivity in pediatric glioblastoma cells. Childs Nerv. Syst..

[36] Liu C., Banister C., Buckhaults P. (2019). Spindle assembly checkpoint inhibition can resensitize p53-null stem cells to cancer chemotherapy. Cancer Res..

[37] Siemeister G., Mengel A., Fernández-Montalván A.E., Bone W., Schröder J., Zitzmann-Kolbe S., Briem H., Prechtl S., Holton S.J., Mönning U., von Ahsen O., Johanssen S., Cleve A., Pütter V., Hitchcock M. (2019). Inhibition of BUB1 kinase by BAY 1816032 sensitizes tumor cells toward taxanes, ATR, and PARP inhibitors in vitro and in vivo. Clin. Cancer Res..

[38] Li Y., Zhuang H., Zhang X., Li Y., Liu Y., Yi X., Qin G., Wei W., Chen R. (2018). Multiomics integration reveals the landscape of prometastasis metabolism in hepatocellular carcinoma. Mol. Cell. Proteomics.

