# Supplementary material for: Dual-functional significance of ATM-mediated phosphorylation of spindle assembly checkpoint component Bub3 in mitosis and the DNA damage response
Source: J Biol Chem. 2022 Jan 25;298(3):101632. doi: 10.1016/j.jbc.2022.101632 (PMC8861116; doi:10.1016/j.jbc.2022.101632)
Supplement: Supplemental Table S1 and Figures S1–S6 [file mmc1.pdf]

Supplementary Table S1. The list of all substrates of ATM in mitosis by SILAC

Protein ID	function	RepID	EntrezID	Accession Number	Molecular Weight
40S ribosomal protein S11	poly (a) rna binding	RS11_HUMAN	6205	P62280	18 kDa
40S ribosomal protein S7	rna binding	RS7_HUMAN	6201	P62081	22 kDa
Keratin, type I cytoskeletal 9	part of cytoskeleton	K1C9_HUMAN	3857	P35527	62 kDa
Serum albumin		ALBU_BOVIN		P02769	69 kDa
L-lactate dehydrogenase B chain	pyruvate->lactate	LDHB_HUMAN	3945	P07195	37 kDa
60S ribosomal protein L10a	rna binding	RL10A_HUMAN	728979	P62906	25 kDa
Transgelin-2	cell differentiation	TAGL2_HUMAN	8407	P37802	22 kDa
60S ribosomal protein L38	rna binding	RL38_HUMAN	6169	P63173	8 kDa
Histone-arginine methyltransferase CARM1	Methylator of DNA	CARM1_HUMAN	10498	Q86X55	66 kDa
60S ribosomal protein L17	poly (a) rna binding	RL17_HUMAN	100132742	P18621	21 kDa
40S ribosomal protein S25	rna binding	RS25_HUMAN	100131196	P62851	14 kDa
DnaJ homolog subfamily C member 8	rna splicing	DNJC8_HUMAN	22826	O75937	30 kDa
Keratin, type II cytoskeletal 6A	structural cytoskeleton	K2C6A_HUMAN	3853	P02538	60 kDa
Ras-related protein Rab-1A	endosome trafficking	RAB1A_HUMAN	5861	P62820	23 kDa
10 kDa heat shock protein, mitochondrial	atp bining, rna binding	CH10_HUMAN	3336	P61604	11 kDa
60S ribosomal protein L34	rna binding	RL34_HUMAN	6164	P49207	13 kDa
Growth factor receptor-bound protein 2	PK binding activity	GRB2_HUMAN	2885	P62993	25 kDa
60S ribosomal protein L28	rna binding	H0YKD8_HUMAN	0	H0YKD8	19 kDa
60S ribosomal protein L9	rna binding	RL9_HUMAN	654350	P32969	22 kDa
Programmed cell death protein 2-like	cell cycle	PDD2L_HUMAN	84306	Q9BRP1	39 kDa
60S ribosomal protein L23a	Nt binding rRNA binding	RL23A_HUMAN	391825	P62750	18 kDa
NADH dehydrogenase [ubiquinone] 1 alpha subcomplex subun	protein complex binding	NDUA4_HUMAN	4697	O00483	9 kDa
Ashwin	embrionic morphogenesis	ASHWN_HUMAN	79074	Q9BVC5	26 kDa
60S ribosomal protein L29	rna binding	RL29_HUMAN	283412	P47914 (+1)	18 kDa
Clathrin light chain B	peptide binding	CLCB_HUMAN	1212	P09497	25 kDa
Zinc finger protein 207	sac checkpoint/bub3	ZN207_HUMAN	7756	O43670	51 kDa
DnaJ homolog subfamily C member 10	hsp70 protein binding	DJC10_HUMAN	54431	Q8IXB1	91 kDa
40S ribosomal protein S23	rna binding	RS23_HUMAN	6228	P62266	16 kDa
Cytoplasmic FMR1-interacting protein 1	actin filament/rac binding	CYFP1_HUMAN	23191	Q7L576	145 kDa
Crk-like protein	sh3/sh2 adaptor activity	CRKL_HUMAN	1399	P46109	34 kDa
14-3-3 protein zeta/delta	pS/pT recognition binding	1433Z_HUMAN	7534	P63104	28 kDa
Protein disulfide-isomerase	protein modifier	PDIA1_HUMAN	5034	P07237	57 kDa
5'-AMP-activated protein kinase subunit gamma-1	atp/adp/amp binding	F8VYY9_HUMAN	0	F8VYY9 (+2)	32 kDa
Mitotic checkpoint protein BUB3	spindle-assembly checkpoint	BUB3_HUMAN	9184	O43684	37 kDa
Heterogeneous nuclear ribonucleoproteins C1/C2	dna/rna binding	HNRPC_HUMAN	3183	P07910	34 kDa
RNA-binding protein Raly	rna binding	RALY_HUMAN	22913	Q9UKM9	32 kDa
40S ribosomal protein S19	rna binding	RS19_HUMAN	6223	P39019	16 kDa
Peptidyl-prolyl cis-trans isomerase B	peptide binding	PPIB_HUMAN	5479	P23284	24 kDa
60 kDa heat shock protein, mitochondrial	dna/rna binding	CH60_HUMAN	644745	P10809	61 kDa
WD repeat-containing protein 5	histone modification	WDR5_HUMAN	11091	P61964	37 kDa
Collagen alpha-2(IV) chain	ecm constituent	CO4A2_HUMAN	1284	P08572	168 kDa
Thioredoxin	fos/jun in IR	THIO_HUMAN	7295	P10599	12 kDa
PEST proteolytic signal-containing nuclear protein	probably cell cycle regulation	PCNP_HUMAN	57092	Q8WW12	19 kDa
Vesicle-associated membrane protein-associated protein A	vessicle trafficking	VAPA_HUMAN	9218	Q9P0L0	28 kDa
60S ribosomal protein L7a	rna binding	RL7A_HUMAN	152663	P62424	30 kDa
Elongation factor 1-alpha 1	nucleotide binding (parp)	EF1A1_HUMAN	645715	P68104 (+1)	50 kDa
Eukaryotic translation initiation factor 4E type 2	g-cap mRNA binder	IF4E2_HUMAN	9470	O60573	28 kDa
60S ribosomal protein L26	rna binding	RL26_HUMAN	100132547	P61254	17 kDa
Serine-threonine kinase receptor-associated protein	catalyst	STRAP_HUMAN	11171	Q9Y3F4	38 kDa
Casein kinase 2 alpha isoform	atp binding ps/pt kinase	Q2I0Y7_HUMAN	1457	Q2I0Y7	45 kDa
Protein enabled homolog	cytoskeleton remodeling	ENAH_HUMAN	55740	Q8N8S7	67 kDa
Nascent polypeptide-associated complex subunit alpha	DNA binding JUN coactivator	NACA_HUMAN	4666	Q13765	23 kDa
Tubulin beta chain	microtubules	TBB5_HUMAN	442308	P07437	50 kDa
Protein LSM12 homolog	??	LSM12_HUMAN	124801	Q3MHD2	22 kDa
Pre-mRNA-processing factor 19	DSB repair	PRP19_HUMAN	27339	Q9UMS4	55 kDa
Cell migration-inducing protein 17	translation initiation	A1KYQ7_HUMAN	0	A1KYQ7 (+1)	105 kDa
Poly(rC)-binding protein 2	nucleotide binding	PCBP2_HUMAN	5094	Q15366	39 kDa
HEAT repeat-containing protein 5B	??	HTR5B_HUMAN	54497	Q9P2D3	224 kDa
60S ribosomal protein L5	rna binding	RL5_HUMAN	388907	P46777	34 kDa
Dedicator of cytokinesis protein 9	activates CDC42	DOCK9_HUMAN	23348	Q9BZ29	236 kDa
Heterogeneous nuclear ribonucleoprotein D-like	transcriptional regulator	HNRDL_HUMAN	9987	O14979	46 kDa
Leucine-rich repeat-containing protein 59	nuclear transport protein	LRC59_HUMAN	55379	Q96AG4	35 kDa
Insulin-like growth factor 2 mRNA-binding protein 3	actin filament/igf2 binding	IF2B3_HUMAN	10643	O00425	64 kDa
Splicing factor 3A subunit 1	rna binding	SF3A1_HUMAN	10291	Q15459	89 kDa
60S acidic ribosomal protein P0	rna binding	RLA0_HUMAN	122589	P05388	34 kDa
RNA-binding motif protein, X chromosome	rna binding	RBMX_HUMAN	27316	P38159	42 kDa
Protein transport protein Sec24D	er trafficking	SC24D_HUMAN	9871	O94855	113 kDa
Ras GTPase-activating protein-binding protein 2	scaffold protien mRNA transport	G3BP2_HUMAN	9908	Q9UN86	54 kDa
Eukaryotic translation initiation factor 2 subunit 3	translation component	IF2G_HUMAN	1968	P41091	51 kDa
Probable E3 ubiquitin-protein ligase MYCBP2	e3 ubiquitin ligase	MYCB2_HUMAN	23077	O75592	510 kDa
PIH1 domain-containing protein 1	epithelial cell differentiation	PIHD1_HUMAN	55011	Q9NWS0	32 kDa
Triosephosphate isomerase	metabolism	TPIS_HUMAN	7167	P60174	31 kDa
Heterogeneous nuclear ribonucleoprotein D0	rna binding	HNRPD_HUMAN	3184	Q14103	38 kDa
Heterogeneous nuclear ribonucleoprotein A1	translational regulator	ROA1_HUMAN	728643	P09651	39 kDa
Eukaryotic translation initiation factor 3 subunit J	translation component	EIF3J_HUMAN	8669	O75822	29 kDa
Eukaryotic translation initiation factor 3 subunit G	translation intitiation	EIF3G_HUMAN	8666	O75821	36 kDa
Interleukin enhancer-binding factor 2	promotes DPK complex form	ILF2_HUMAN	3608	Q12905	43 kDa
RNA-binding motif, single-stranded-interacting protein 1	rna binding	RBMS1_HUMAN	5937	P29558	45 kDa
Cleavage and polyadenylation specificity factor subunit	mRNA export	CPSF5_HUMAN	11051	O43809	26 kDa
Splicing factor 45	rna binding	SPF45_HUMAN	84991	Q96I25	45 kDa
Trinucleotide repeat-containing gene 6A protein	miRNA silencing pathways	TNR6A_HUMAN	27327	Q8NDV7	210 kDa
Heterogeneous nuclear ribonucleoprotein R	rna binding	HNRPR_HUMAN	10236	O43390	71 kDa
Caprin-1	rna binding	CAPR1_HUMAN	4076	Q14444	78 kDa
RNA-binding protein Musashi homolog 2	rna binding	MSI2H_HUMAN	124540	Q96DH6	35 kDa
Nucleophosmin	centrosome duplication	NPM_HUMAN	729686	P06748	33 kDa
Clathrin light chain A	vessicle coating	CLCA_HUMAN	1211	P09496 (+1)	27 kDa
Nucleoporin NUP188 homolog	mitotic nuclear envelope assembly	NU188_HUMAN	23511	Q5SRE5	196 kDa
Probable ATP-dependent RNA helicase DDX6	mRNA degradation	DDX6_HUMAN	1656	P26196	54 kDa
60S ribosomal protein L21	rna binding	RL21_HUMAN	653156	P46778	19 kDa
Splicing factor 3B subunit 4	rna binding	SF3B4_HUMAN	10262	Q15427	44 kDa
Keratin, type I cytoskeletal 16	structural cytoskeleton	K1C16_HUMAN	3868	P08779	51 kDa
E3 ubiquitin-protein ligase TRIM32	e3 ubiquitin ligase	TRI32_HUMAN	22954	Q13049	72 kDa
ADP-ribosylation factor GTPase activating protein 1		B7ZBI2_HUMAN	0	B7ZBI2	44 kDa
TAR DNA-binding protein 43	nucleotide binding	TADBP_HUMAN	23435	Q13148	45 kDa
Cleavage and polyadenylation specificity factor subunit	mRNA processing	CPSF3_HUMAN	51692	Q9UKF6	77 kDa
Ras-related protein Rab-5C	protein transport	RAB5C_HUMAN	5878	P51148	23 kDa
40S ribosomal protein S17-like	rna binding	RS17L_HUMAN	0	P0CW22	16 kDa
40S ribosomal protein S9	rna binding	RS9_HUMAN	6203	P46781	23 kDa
Bcl-2-associated transcription factor 1	apoptosis (dna damage induced)	E9PQN2_HUMAN	0	E9PQN2 (+1)	86 kDa
RNA-binding protein FUS	genome integrity maintenance	FUS_HUMAN	2521	P35637 (+1)	53 kDa
Cyclin-dependent kinase 12	regulates DDR proteins/genomic stability	CDK12_HUMAN	0	Q9NYV4	164 kDa
PABPC4 protein	rna binding	Q4VC03_HUMAN	8761	Q4VC03	72 kDa
Splicing factor U2AF 65 kDa subunit	pre-rna splicing	U2AF2_HUMAN	11338	P26368	54 kDa
Plasminogen activator inhibitor 1 RNA-binding protein	rna binding	PAIRB_HUMAN	26135	Q8NC51	45 kDa
WD repeat-containing protein 61	histone modification	WDR61_HUMAN	80349	Q9GZS3	34 kDa
60S ribosomal protein L8	rna binding	E9PKZ0_HUMAN	0	E9PKZ0 (+1)	22 kDa
28 kDa heat- and acid-stable phosphoprotein	pdgfa stimulated cell growth	HAP28_HUMAN	645181	Q13442	21 kDa
60S ribosomal protein L19	rna binding	RL19_HUMAN	100129424	P84098	23 kDa

60S ribosomal protein L3	rna binding	RL3_HUMAN	6122	P39023	46 kDa
Eukaryotic translation initiation factor 3 subunit I	translation intitiation	EIF3I_HUMAN	8668	Q13347	37 kDa
Clathrin interactor 1	vessicle coating	B7Z6F8_HUMAN	0	B7Z6F8	70 kDa
Interferon-inducible double stranded RNA-dependent prote	required for siRNA production by DICER	PRKRA_HUMAN	8575	O75569	34 kDa
Dynamin-2	microtubule binding	DYN2_HUMAN	1785	P50570	98 kDa
40S ribosomal protein S3	rna binding	RS3_HUMAN	440991	P23396	27 kDa
60S ribosomal protein L23	rna binding	RL23_HUMAN	646949	P62829	15 kDa
Splicing factor 3B subunit 5	rna splicing	SF3B5_HUMAN	83443	Q9BWJ5	10 kDa
Splicing factor 3B subunit 1	rna processing	SF3B1_HUMAN	23451	O75533	146 kDa
Filamin-A	actin filament binding	FLNA_HUMAN	2316	P21333	281 kDa
Splicing factor 3A subunit 2	rna binding	SF3A2_HUMAN	8175	Q15428	49 kDa
Transcription elongation factor SPT4	transcriptional regulator	SPT4H_HUMAN	6827	P63272	13 kDa
Polypyrimidine tract-binding protein 1	pre-mRNA splicing	PTBP1_HUMAN	5725	P26599 (+1)	57 kDa
Cell division cycle and apoptosis regulator protein 1	regulates myc ccnb1 and cdkn1a cell cycle	CCAR1_HUMAN	55749	Q8IX12	133 kDa
Proliferation-associated protein 2G4	erbb4 regulated signaling pathway	PA2G4_HUMAN	647033	Q9UQ80	44 kDa
Splicing factor 3A subunit 3	rna splicing	SF3A3_HUMAN	10946	Q12874	59 kDa
Agrin	ecm constituent	AGRIN_HUMAN	375790	O00468	215 kDa
Vacuolar protein sorting-associated protein 13C		VP13C_HUMAN	54832	Q709C8	422 kDa
40S ribosomal protein S12	rna binding	RS12_HUMAN	727997	P25398	15 kDa
Pre-mRNA-processing factor 40 homolog A	rna splicing	PR40A_HUMAN	55660	O75400	109 kDa
mRNA cap guanine-N7 methyltransferase	mRNA translocation	MCES_HUMAN	8731	O43148	55 kDa
Ubiquitin-associated protein 2-like	rna binding	UBP2L_HUMAN	9898	Q14157	115 kDa
Cell differentiation protein RCD1 homolog	mRNA deadenylase	B7Z1E5_HUMAN	0	B7Z1E5 (+1)	37 kDa
Regulator of nonsense transcripts 1	rna dependent helicase	RENT1_HUMAN	5976	Q92900	124 kDa
Eukaryotic translation initiation factor 3 subunit K	translation intitiation	EIF3K_HUMAN	27335	Q9UBQ5	25 kDa
Nuclear pore complex protein Nup214	mitotic nuclear envelope disassembly	NU214_HUMAN	8021	P35658	214 kDa
Cell growth-inhibiting protein 34	translation	Q08ES8_HUMAN	6135	Q08ES8	20 kDa
28S ribosomal protein S7, mitochondrial	rna binding	RT07_HUMAN	51081	Q9Y2R9	28 kDa
40S ribosomal protein S6	rna binding	RS6_HUMAN	729389	P62753	29 kDa
Ras-related protein Rab-11A	major regulator of during cytokenesis	RB11A_HUMAN	8766	P62491 (+3)	24 kDa
YLP motif-containing protein 1	regulator of TERT activity	YLPM1_HUMAN	56252	P49750	220 kDa
GTP-binding nuclear protein Ran	mitotic spindle formation (w/birc5/survivin)	RAN_HUMAN	5901	P62826	24 kDa
Eukaryotic initiation factor 4A-I	g-cap mRNA binder	IF4A1_HUMAN	1973	P60842	46 kDa
40S ribosomal protein S26	rna binding	RS26_HUMAN	392256	P62854 (+1)	13 kDa
Heterogeneous nuclear ribonucleoprotein A/B	mRNA biniding	ROAA_HUMAN	3182	Q99729	36 kDa
Double-stranded RNA-binding protein Staufen homolog 1	dsRNA binding mRNA localization	STAU1_HUMAN	6780	O95793	63 kDa
Superkiller viralicidic activity 2-like 2	pre-mRNA splicing	SK2L2_HUMAN	23517	P42285	118 kDa
Histone H1x	condensation of nucleosome chains	H1X_HUMAN	8971	Q92522	22 kDa
Serine/threonine-protein kinase WNK1	electrolyte homeostasis	WNK1_HUMAN	100132369	Q9H4A3	251 kDa
UPF0568 protein C14orf166		CN166_HUMAN	51637	Q9Y224	28 kDa
RuvB-like 2	chromatin remodeling replicative senescence	RUVB2_HUMAN	10856	Q9Y230	51 kDa
Ribonucleoprotein PTB-binding 1	rna binding	RAVR1_HUMAN	125950	Q8IY67	64 kDa
Fatty acid synthase	fatty acid sythensis	FAS_HUMAN	2194	P49327	273 kDa
Nuclease-sensitive element-binding protein 1	transcription regulator binds RNA	YBOX1_HUMAN	4904	P67809	36 kDa
Cyclin-dependent kinase 1	cell cycle regulator G2-M transition	CDK1_HUMAN	983	P06493	34 kDa
Heat shock 70 kDa protein 1A/1B	protein stabilization	HSP71_HUMAN	3304	P08107	70 kDa
Guanine nucleotide-binding protein subunit beta-2-like 1	G0/G1 prolonger	GBLP_HUMAN	10399	P63244	35 kDa
SEC23-interacting protein	protein transport	S23IP_HUMAN	11196	Q9Y6Y8	111 kDa
Mediator of RNA polymerase II transcription subunit 13-l	transcriptional regulator	MD13L_HUMAN	23389	Q71F56	243 kDa
mRNA export factor	rna binding	RAE1L_HUMAN	8480	P78406 (+1)	41 kDa
RNA 3'-terminal phosphate cyclase	rna processing	RTC1_HUMAN	8634	O00442	39 kDa
Filamin-C	muscle specific	FLNC_HUMAN	2318	Q14315	291 kDa
Polyadenylate-binding protein 1	rna binding	PABP1_HUMAN	341315	P11940	71 kDa
RPL14 protein	part of ribosome	Q6IPH7_HUMAN	9045	Q6IPH7	24 kDa
RuvB-like 1	chromatin remodeling DNA replication	RUVB1_HUMAN	8607	Q9Y265	50 kDa
Regulation of nuclear pre-mRNA domain-containing protein	CDK transcription regulator	RPR1B_HUMAN	58490	Q9NQG5	37 kDa
Ras GTPase-activating protein-binding protein 1	rna binding	G3BP1_HUMAN	10146	Q13283 (+1)	52 kDa
Mediator of RNA polymerase II transcription subunit 8	protein modification	MED8_HUMAN	112950	Q96G25	29 kDa
Paraspeckle component 1	transcription regulator	PSPC1_HUMAN	642395	Q8WXF1	59 kDa
ATP-dependent RNA helicase DDX42	TP53BP2 interactor	DDX42_HUMAN	11325	Q86XP3	103 kDa
Peroxiredoxin-1	redox regulation	PRDX1_HUMAN	5052	Q06830	22 kDa
tRNA-splicing ligase RtcB homolog	rna splicing ligase	RTCB_HUMAN	51493	Q9Y3I0	55 kDa
SCY1-like protein 2	negative regulator of Wnt signaling	SCYL2_HUMAN	55681	Q6P3W7	104 kDa
Symplekin	cell adhesion	SYMPK_HUMAN	8189	Q92797	141 kDa
Interleukin enhancer-binding factor 3	nucleotide binding	ILF3_HUMAN	3609	Q12906	95 kDa
WD repeat and FYVE domain-containing protein 3	selective autophagy	WDFY3_HUMAN	23001	Q8IZQ1	395 kDa
Zinc finger protein 326	nucleotide binding	ZN326_HUMAN	284695	Q5BKZ1	66 kDa
AH receptor-interacting protein		AIP_HUMAN	9049	O00170	38 kDa
La-related protein 4	cell morphology and cytoskeletal organization	LARP4_HUMAN	113251	Q71RC2	81 kDa
Zinc finger SWIM domain-containing protein KIAA0913	zinc ion binding	K0913_HUMAN	23053	A7E2V4	197 kDa
40S ribosomal protein S4, X isoform	rna binding	RS4X_HUMAN	391777	P62701	30 kDa
Eukaryotic translation initiation factor 3 subunit F	translation intitiation	EIF3F_HUMAN	390282	O00303	38 kDa
Clathrin heavy chain 1	vessicle coating	CLH1_HUMAN	1213	Q00610	192 kDa
40S ribosomal protein S2	mRNA biniding	RS2_HUMAN	286444	P15880	31 kDa
Zinc finger FYVE domain-containing protein 26	cytokenesis	ZFY26_HUMAN	23503	Q68DK2	285 kDa
Ankyrin repeat and KH domain-containing protein 1	anti-apoptotic protein	ANKH1_HUMAN	54882	Q8IWZ3	269 kDa
Sorting nexin-9	progress through mitosis and cytokinesis	SNX9_HUMAN	51429	Q9Y5X1	67 kDa
CIP29 protein	nuclear protein	Q567R9_HUMAN	84324	Q567R9	17 kDa
Heterogeneous nuclear ribonucleoprotein U	nucleotide binding	HNRPU_HUMAN	3192	Q00839	91 kDa
Fibronectin	ECM	FINC_HUMAN	2335	P02751	263 kDa
Heterogeneous nuclear ribonucleoprotein U-like protein 1	transcriptional regulator	HNRL1_HUMAN	11100	Q9BUJ2	96 kDa
Heterogeneous nuclear ribonucleoprotein A0	RNA binding	ROA0_HUMAN	10949	Q13151	31 kDa
PERQ amino acid-rich with GYF domain-containing protein	tyrosine kinase receptor signaling regulator	PERQ2_HUMAN	26058	Q6Y7W6	150 kDa
WD repeat-containing protein mio	gator2 complex regulator of mTOR	MIO_HUMAN	54468	Q9NXC5	99 kDa
U1 small nuclear ribonucleoprotein C	rna splicing	RU1C_HUMAN	6631	P09234	17 kDa
Serine/threonine-protein kinase mTOR	hub protein	MTOR_HUMAN	2475	P42345	289 kDa
Nuclear fragile X mental retardation-interacting protein	rna binding	NUFP2_HUMAN	57532	Q7Z417	76 kDa
60S ribosomal protein L4	rna binding	E7EWF1_HUMAN	0	E7EWF1	46 kDa
Putative ATP-dependent RNA helicase DHX30	associates with mitocondrial dna	DHX30_HUMAN	22907	Q7L2E3	134 kDa
DmX-like protein 1		DMXL1_HUMAN	1657	Q9Y485	338 kDa
LRCH2 protein		Q08AD5_HUMAN	57631	Q08AD5 (+1)	83 kDa
40S ribosomal protein S8	rna binding	RS8_HUMAN	6202	P62241	24 kDa
Src substrate cortactin	cytoskeleton remodeling	SRC8_HUMAN	2017	Q14247	62 kDa
14-3-3 protein epsilon	non-specific regulation	1433E_HUMAN	440917	P62258	29 kDa
REST corepressor 1	H3 Demethylation Chromatin binding	RCOR1_HUMAN	23186	Q9UKL0	53 kDa
Creatine kinase U-type, mitochondrial	energy transduction	KCRU_HUMAN	548596	P12532	47 kDa
Phosphatidylinositol 4-kinase alpha	atp binding	PI4KA_HUMAN	5297	P42356	231 kDa
Protein LSM14 homolog A	poly(A) RNA binding	LS14A_HUMAN	26065	Q8ND56	51 kDa
Calcium homeostasis endoplasmic reticulum protein	calcium homeostasis	CHERP_HUMAN	10523	Q8IWX8	104 kDa
Small nuclear ribonucleoprotein Sm D2	splicing mRNA	SMD2_HUMAN	119358	P62316	14 kDa
Probable ubiquitin carboxyl-terminal hydrolase FAF-X	regulates chromosome alignment and segregation	USP9X_HUMAN	8239	Q93008	292 kDa
Mediator of RNA polymerase II transcription subunit 20	DNA directed RNA Polymerase	MED20_HUMAN	9477	Q9H944	23 kDa
Keratin, type II cytoskeletal 5		K2C5_HUMAN	3852	P13647	62 kDa
40S ribosomal protein S13	rna binding	RS13_HUMAN	645630	P62277	17 kDa
40S ribosomal protein S27	rna binding	RS27_HUMAN	100132488	P42677	9 kDa
Heterogeneous nuclear ribonucleoprotein K transcript var	RNA Binding	Q5EC54_HUMAN	644063	Q5EC54	51 kDa
60S ribosomal protein L12	rna binding	RL12_HUMAN	6136	P30050	18 kDa
Tubulin alpha-1A chain	major consituent of microtubules	TBA1A_HUMAN	7846	Q71U36	50 kDa
Putative RNA-binding protein Luc7-like 1	RNA Binding	LUC7L_HUMAN	55692	Q9NQ29	44 kDa
Ewing sarcoma breakpoint region 1	transcriptional repressor	B0QYK1_HUMAN	2130	B0QYK1 (+3)	63 kDa
Protein PRRC2C	poly(A) RNA binding	PRC2C_HUMAN	23215	Q9Y520	317 kDa
40S ribosomal protein S20	Rna Binding	RS20_HUMAN	6224	P60866	13 kDa
Signal transducer and activator of transcription 3	signal transducer	STAT3_HUMAN	6774	P40763	88 kDa

Pre-mRNA-processing-splicing factor 8	splicing mRNA	PRP8_HUMAN	10594	Q6P2Q9	274 kDa
Mediator of RNA polymerase II transcription subunit 22	DNA directed RNA Polymerase	MED22_HUMAN	6837	Q15528 (+1)	22 kDa
Complement component 1 Q subcomponent-binding protein, m	hub protein	C1QBP_HUMAN	708	Q07021	31 kDa
Eukaryotic translation initiation factor 3 subunit E	translation initiator	EIF3E_HUMAN	3646	P60228	52 kDa
Dihydropyrimidinase-related protein 2	cell migration	DPYL2_HUMAN	1808	Q16555	62 kDa
DNA-directed RNA polymerase II subunit RPB2	DNA directed RNA Polymerase	RPB2_HUMAN	5431	P30876	134 kDa
Protein PRRC2B	poly(A) RNA binding	PRC2B_HUMAN	84726	Q5JSZ5	243 kDa
Constitutive coactivator of PPAR-gamma-like protein 1	oxidative stress-induced survival signaling	F120A_HUMAN	23196	Q9NZB2	122 kDa
CCR4-NOT transcription complex subunit 1	mRNA degradation	CNOT1_HUMAN	23019	A5YKK6	267 kDa
U1 small nuclear ribonucleoprotein 70 kDa	assembly of the spliceosome	RU17_HUMAN	6625	P08621	52 kDa
78 kDa glucose-regulated protein	protein folding	GRP78_HUMAN	400750	P11021	72 kDa
Putative RNA-binding protein Luc7-like 2	poly(A) RNA binding	LC7L2_HUMAN	51631	Q9Y383	47 kDa
Desmoplakin	cytoskeleton component	DESP_HUMAN	1832	P15924	332 kDa
Heterogeneous nuclear ribonucleoprotein M	receptor for carcinoembryonic antigen	HNRPM_HUMAN	4670	P52272	78 kDa
Probable ATP-dependent RNA helicase DDX5	alternative regulation of pre-mRNA splicing	DDX5_HUMAN	1655	P17844	69 kDa
DNA-directed RNA polymerases I, II, and III subunit RPAB	dna binding	RPAB1_HUMAN	5434	P19388	25 kDa
Protein transport protein Sec31A	coating of vesicles from ER	SC31A_HUMAN	22872	O94979	133 kDa
Eukaryotic translation initiation factor 3 subunit H	translation initiator	EIF3H_HUMAN	8667	O15372 (+1)	40 kDa
E3 SUMO-protein ligase RanBP2	nuclear export pathway.	RBP2_HUMAN	5903	P49792	358 kDa
60S ribosomal protein L30	poly(A) RNA binding	RL30_HUMAN	6156	P62888	13 kDa
ATP-dependent RNA helicase DDX3X	nucleic acid binding	B5BTY4_HUMAN	0	B5BTY4 (+1)	73 kDa
U1 small nuclear ribonucleoprotein A	RNA splicing	SNRPA_HUMAN	6626	P09012	31 kDa
Heterogeneous nuclear ribonucleoprotein L	Apex1 associated and RNA binding	HNRPL_HUMAN	3191	P14866	64 kDa
Peroxiredoxin-6	redox regulation	PRDX6_HUMAN	9588	P30041	25 kDa
Cold-inducible RNA-binding protein	rna binding	CIRBP_HUMAN	1153	Q14011	19 kDa
5'-3' exoribonuclease 1	mRNA degradation	XRN1_HUMAN	54464	Q8IZH2	194 kDa
Importin subunit alpha-2	adapter protein for nuclear receptor KPNB1	IMA2_HUMAN	3838	P52292 (+1)	58 kDa
Protein arginine N-methyltransferase 1	Arginine methyltransferase	ANM1_HUMAN	3276	Q99873	42 kDa
CAD protein	the pyrimidine pathway	PYR1_HUMAN	790	P27708	243 kDa
CCR4-NOT transcription complex subunit 7	anti-proliferative activity mrna degradation	G3V108_HUMAN	0	G3V108 (+2)	28 kDa
Elongation factor 1-delta	translation	EF1D_HUMAN	1936	P29692 (+1)	31 kDa
Cleavage stimulation factor subunit 3	pre mRNA splicing	CSTF3_HUMAN	1479	Q12996	83 kDa
Pre-mRNA branch site protein p14	pre-mRNA splicing	PM14_HUMAN	51639	Q9Y3B4	15 kDa
Ribosomal protein S27a	ribosome component	Q5RKT7_HUMAN	728590	Q5RKT7	18 kDa
Splicing factor 3B subunit 3	nucleic acid binding	SF3B3_HUMAN	23450	Q15393	136 kDa
PHD finger-like domain-containing protein 5A	transcription factor	PHF5A_HUMAN	84844	Q7RTV0	12 kDa
Protein transport protein Sec24C	coating of vesicles from ER	SC24C_HUMAN	9632	P53992	118 kDa
Eukaryotic translation initiation factor 4 gamma 1	translation initiator	IF4G1_HUMAN	1981	Q04637	175 kDa
Spectrin alpha chain, brain	calcium regulator cytoskeleton remodeling	SPTA2_HUMAN	6709	Q13813	285 kDa
Putative pre-mRNA-splicing factor ATP-dependent RNA heli	disassembly of spliceosomes	DHX15_HUMAN	1665	O43143	91 kDa
ATP-binding cassette sub-family E member 1	regulation of mRNA turnover	ABCE1_HUMAN	6059	P61221	67 kDa
E3 ubiquitin-protein ligase RNF213	angiogenesis	RN213_HUMAN	57674	Q63HN8	591 kDa
Mitochondrial import inner membrane translocase subunit	part of tim23 complex	TIM50_HUMAN	92609	Q3ZCQ8	40 kDa
Citron	regulates cytokenesis	Q2M5E1_HUMAN	11113	Q2M5E1	237 kDa
ELAV-like protein 1	RNA binding	ELAV1_HUMAN	1994	Q15717	36 kDa
Heterogeneous nuclear ribonucleoprotein U-like protein 2	poly(A) RNA binding	HNRL2_HUMAN	221092	Q1KMD3	85 kDa
Serine/threonine-protein kinase SMG1	genotoxic stress-activated protein kinase	SMG1_HUMAN	23049	Q96Q15	410 kDa
Protein FAM98A	poly(A) RNA binding	FA98A_HUMAN	25940	Q8NCA5	55 kDa
DAZ-associated protein 1	rna binding protein	DAZP1_HUMAN	26528	Q96EP5	43 kDa
ATP-dependent RNA helicase DDX1	transcriptional regulator of cyclin CCND2	DDX1_HUMAN	1653	Q92499	82 kDa
Ankyrin repeat domain-containing protein 17	nothing important	ANR17_HUMAN	26057	O75179	274 kDa
40S ribosomal protein S5	rna binding	RS5_HUMAN	6193	P46782	23 kDa
Probable ATP-dependent RNA helicase DDX17	RNA-dependent ATPase activity	DDX17_HUMAN	10521	Q92841	80 kDa
Ataxin-2-like protein	poly(A) RNA binding	ATX2L_HUMAN	11273	Q8WWM7	113 kDa
THO complex subunit 4	nucleotide Binding	THOC4_HUMAN	10189	Q86V81	27 kDa
Heterogeneous nuclear ribonucleoproteins A2/B1	pre mRNA splicing	ROA2_HUMAN	3181	P22626	37 kDa
U5 small nuclear ribonucleoprotein 200 kDa helicase	pre-mRNA splicing	U520_HUMAN	23020	O75643	245 kDa
G patch domain-containing protein 8		GPTC8_HUMAN	23131	Q9UKJ3	164 kDa
Small nuclear ribonucleoprotein E	rna processing	RUXE_HUMAN	6635	P62304	11 kDa
Mediator of RNA polymerase II transcription subunit 1	Chromatin Binding	MED1_HUMAN	5469	Q15648	168 kDa
Transmembrane emp24 domain-containing protein 10	vesicular protein trafficking	TMEDA_HUMAN	10972	P49755	25 kDa
Poly(rC)-binding protein 1	nucleotide binding	PCBP1_HUMAN	5093	Q15365	37 kDa
CDKN2A-interacting protein	Activates p53/TP53 by CDKN2A-dependent	CARF_HUMAN	55602	Q9NXV6	61 kDa
Ubiquitin-associated protein 2	poly(A) RNA binding	UBAP2_HUMAN	55833	Q5T6F2	117 kDa
Heterogeneous nuclear ribonucleoprotein Q	nucleotide binding	HNRPQ_HUMAN	10492	O60506	70 kDa
Serine/threonine-protein phosphatase PP1-alpha catalytic	regulates NEK2	PP1A_HUMAN	5499	P62136	38 kDa
Serine/arginine-rich splicing factor 1	mRNA nuclear export	SRSF1_HUMAN	6426	Q07955	28 kDa
Heterogeneous nuclear ribonucleoprotein H	pre-mRNA splicing	HNRH1_HUMAN	3187	P31943	49 kDa
Protein SEC13 homolog	component of nuclear pore complex	SEC13_HUMAN	6396	P55735	36 kDa
ATP synthase subunit g, mitochondrial	H+ transporter	E9PN17_HUMAN	0	E9PN17 (+1)	8 kDa
Peroxisomal multifunctional enzyme type 2	beta oxidation pathway	DHB4_HUMAN	3295	P51659	80 kDa
Heat shock cognate 71 kDa protein	repressor of transcriptional activation	HSP7C_HUMAN	3312	P11142	71 kDa
Melanoma inhibitory activity protein 3	negative regulator of cell adhesion/migration	MIA3_HUMAN	375056	Q5JRA6	214 kDa
Spectrin beta chain, brain 1	calcium-dependent movement of the cytoskeleton	SPTB2_HUMAN	6711	Q01082	275 kDa
DNA-binding protein A	Represses GM-CSF promoter	DBPA_HUMAN	8531	P16989	40 kDa
Calponin-2	thin filament associated protein	B4DDF4_HUMAN	0	B4DDF4 (+3)	33 kDa
Small nuclear ribonucleoprotein Sm D1		Q7Z5A3_HUMAN	6632	Q7Z5A3	13 kDa
CREB-binding protein	histone acetylation	CBP_HUMAN	1387	Q92793	265 kDa
Small nuclear ribonucleoprotein G	component of splicosome	RUXG_HUMAN	100130289	P62308	8 kDa
RNA-binding protein 12	nucleotide binding	RBM12_HUMAN	8904	Q9NTZ6	97 kDa
Filamin B	cytoskeletal component	B2ZZ83_HUMAN	2317	B2ZZ83 (+1)	282 kDa
TATA-binding protein-associated factor 2N	nucleotide binding	RBP56_HUMAN	8148	Q92804	62 kDa
Alpha-enolase	Glycolysis	ENOA_HUMAN	2023	P06733	47 kDa
Dedicator of cytokinesis 4	Wnt Signaling pathway	Q149N6_HUMAN	9732	Q149N6 (+1)	225 kDa
Sacsin	regulator of the Hsp70 chaperone machinery	SACS_HUMAN	26278	Q9NZJ4	521 kDa
DNA-directed RNA polymerase II subunit RPB1	transcription dna-->rna	RPB1_HUMAN	5430	P24928	217 kDa
60S ribosomal protein L18	ribosome component	RL18_HUMAN	6141	Q07020	22 kDa
Eukaryotic translation initiation factor 3 subunit M	translation initiator	EIF3M_HUMAN	10480	Q7L2H7	43 kDa
Eukaryotic translation initiation factor 4E	translation regulator	IF4E_HUMAN	100131565	P06730	25 kDa
Serine/arginine repetitive matrix protein 2	pre-mRNA splicing	SRRM2_HUMAN	23524	Q9UQ35	300 kDa
Stress-70 protein, mitochondrial	control of cell proliferation and cellular aging	GRP75_HUMAN	3313	P38646	74 kDa
Putative deoxyribose-phosphate aldolase	biochemical reaction	DEOC_HUMAN	51071	Q9Y315	35 kDa
Insulin receptor substrate 4	IGF1R mitogenic signaling pathway	IRS4_HUMAN	8471	O14654	134 kDa
RNA-binding protein 39	transcriptional coactivator of steroid nuclear recep	RBM39_HUMAN	9584	Q14498	59 kDa
5'-3' exoribonuclease 2	mRNA degradation	XRN2_HUMAN	22803	Q9H0D6	109 kDa
S-phase kinase-associated protein 1	component of SCF, cell cycle progression	SKP1_HUMAN	6500	P63208	19 kDa
Matrin-3	nuclear retention of defective RNAs	MATR3_HUMAN	9782	P43243	95 kDa
U2 small nuclear ribonucleoprotein A'	rna splicing	RU2A_HUMAN	6627	P09661	28 kDa
Far upstream element-binding protein 1	regulates MYC	FUBP1_HUMAN	8880	Q96AE4	68 kDa
Insulin-like growth factor 2 mRNA-binding protein 1	mRNA Binding	IF2B1_HUMAN	10642	Q9NZI8	63 kDa
40S ribosomal protein S3a	ribosome component	RS3A_HUMAN	146053	P61247	30 kDa
60S ribosomal protein L31	ribosome component	B7Z4C8_HUMAN	0	B7Z4C8 (+2)	15 kDa
Nuclear pore complex protein Nup88	nuclear pore complex	NUP88_HUMAN	4927	Q99567	84 kDa
Splicing factor 3B subunit 2	pre-mRNA splicing	SF3B2_HUMAN	10992	Q13435	100 kDa
Palladin	Cytoskeletal protein for actin cytoskeleton	PALLD_HUMAN	23022	Q8WX93	151 kDa
Nuclear pore complex protein Nup155	component of nuclear pore complex	NU155_HUMAN	9631	O75694	155 kDa
Secretory carrier-associated membrane protein 3	Functions in post-Golgi recycling pathways	SCAM3_HUMAN	10067	O14828	38 kDa
60S ribosomal protein L15	ribosome component	RL15_HUMAN	728002	P61313	24 kDa
WD repeat-containing protein 82	PTW/PP1 phosphatase complex mitotic exit	WDR82_HUMAN	80335	Q6UXN9	35 kDa
DNA-dependent protein kinase catalytic subunit	required for double-strand break	PRKDC_HUMAN	731751	P78527	469 kDa
E3 ubiquitin-protein ligase TRIM33	control of cell proliferation	TRI33_HUMAN	51592	Q9UPN9	123 kDa
Keratin, type II cytoskeletal 8		K2C8_HUMAN	390601	P05787	54 kDa
RNA-binding motif, single-stranded-interacting protein 2	Nucleotide binding	RBMS2_HUMAN	5939	Q15434	44 kDa

40S ribosomal protein S14	ribosome component	RS14_HUMAN	6208	P62263	16 kDa
ADP/ATP translocase 2	chromosome segregation part of MMXD complex	ADT2_HUMAN	392301	P05141	33 kDa
Far upstream element-binding protein 3	nucleotide binding	FUBP3_HUMAN	8939	Q96I24	62 kDa
Small nuclear ribonucleoprotein-associated proteins B an	rna splicing	RSMB_HUMAN	6628	P14678 (+3)	25 kDa
Eukaryotic translation initiation factor 3 subunit L	translation initiator	EIF3L_HUMAN	51386	Q9Y262	67 kDa
Rotatin	governs left-right specification	RTTN_HUMAN	25914	Q86VV8	249 kDa
Replication protein A 32 kDa subunit	mitotic G1 Damage Checkpoint	RFA2_HUMAN	6118	P15927	29 kDa
Utrophin	anchoring the cytoskeleton to the plasma membrane.	UTRO_HUMAN	7402	P46939	394 kDa
Nuclear receptor corepressor 1	transcriptional repressor	NCOR1_HUMAN	9611	O75376	270 kDa
Peptidyl-prolyl cis-trans isomerase A	protein folding	Q567Q0_HUMAN	653214	Q567Q0	11 kDa
Cleavage and polyadenylation specificity factor subunit	pre-mRNA 3'-end formation	CPSF4_HUMAN	10898	O95639	30 kDa
Protein Shroom2	actin binding	SHRM2_HUMAN	357	Q13796	176 kDa
Heterogeneous nuclear ribonucleoprotein A3	RNA regulator	ROA3_HUMAN	220988	P51991	40 kDa
Protein transport protein Sec23B	vesicle coating	SC23B_HUMAN	10483	Q15437	86 kDa
Nucleolin	Induces chromosome condensation	NUCL_HUMAN	4691	P19338	77 kDa
Ribose-5-phosphate isomerase	carbohydrate metabolism	RPIA_HUMAN	22934	P49247	33 kDa
Prohibitin	inhibitor of DNA synthesis, proliferation regulator	PHB_HUMAN	5245	P35232	30 kDa
RNA-binding protein PNO1	poly(A) RNA binding	PNO1_HUMAN	56902	Q9NRX1	28 kDa
Chromatin target of PRMT1 protein	mrna export	CHTOP_HUMAN	26097	Q9Y3Y2	26 kDa
Dynein light chain roadblock-type 1	vesicle motility	DLRB1_HUMAN	83658	Q9NP97	11 kDa
Gamma-tubulin complex component 6	spindle pole component	GCP6_HUMAN	85378	Q96RT7	200 kDa
Splicing factor 1	pre-mRNA splicing	SF01_HUMAN	7536	Q15637	68 kDa
Tyrosine-protein kinase receptor	activation of MAPK activity	Q8TDJ5_HUMAN	10342	Q8TDJ5	89 kDa
TGF-beta-activated kinase 1 and MAP3K7-binding protein 1	kinase activity activator	TAB1_HUMAN	10454	Q15750	55 kDa
Small nuclear ribonucleoprotein Sm D3	rna splicing	SMD3_HUMAN	6634	P62318	14 kDa
Cytoplasmic dynein 1 heavy chain 1	cellular motility	DYHC1_HUMAN	1778	Q14204	532 kDa
Afadin	adhesion system	AFAD_HUMAN	730031	P55196	207 kDa
Cold shock domain-containing protein E1	mrna turnover	E9PGZ0_HUMAN	0	E9PGZ0 (+1)	91 kDa
ATP-dependent RNA helicase A	atp binding	DHX9_HUMAN	1660	Q08211	141 kDa
Eukaryotic translation initiation factor 3 subunit A	translation initiator	EIF3A_HUMAN	8661	Q14152 (+1)	167 kDa
Heterogeneous nuclear ribonucleoprotein F	pre-mRNA splicing	HNRPF_HUMAN	3185	P52597	46 kDa
60S acidic ribosomal protein P2	elongation protein synthesis	RLA2_HUMAN	6181	P05387	12 kDa
YTH domain family protein 3	rna binding	YTHD3_HUMAN	253943	Q7Z739	64 kDa
Mediator of RNA polymerase II transcription subunit 4	Component of the Mediator complex	MED4_HUMAN	29079	Q9NPJ6	30 kDa
Vimentin	intermediate filaments	VIME_HUMAN	7431	P08670	54 kDa
28S ribosomal protein S22, mitochondrial	ribosome component	G5E9V5_HUMAN	0	G5E9V5 (+1)	41 kDa
60S ribosomal protein L32	ribosome component	F8W727_HUMAN	0	F8W727 (+1)	18 kDa
Glyceraldehyde-3-phosphate dehydrogenase	hub protein	G3P_HUMAN	100133042	P04406	36 kDa
40S ribosomal protein SA	ribosome component	RSSA_HUMAN	653162	P08865	33 kDa
DNA-directed RNA polymerase II subunit RPB3	nucleotide binding	RPB3_HUMAN	5432	P19387	31 kDa
Probable helicase with zinc finger domain	nucleotide binding	HELZ_HUMAN	9931	P42694	219 kDa
Importin subunit beta-1	nuclear import of H1 Histone	IMB1_HUMAN	3837	Q14974	97 kDa
28S ribosomal protein S2, mitochondrial	ribosome component	RT02_HUMAN	51116	Q9Y399	33 kDa
Far upstream element-binding protein 2	activation of estrogen receptor target genes	FUBP2_HUMAN	8570	Q92945	73 kDa
Histone acetyltransferase p300	Chromatin remodeling	EP300_HUMAN	2033	Q09472	264 kDa
BMP-2-inducible protein kinase	osteoblast differentiation	BMP2K_HUMAN	55589	Q9NSY1	129 kDa
Baculoviral IAP repeat-containing protein 6	mitotic nuclear division	BIRC6_HUMAN	57448	Q9NR09	530 kDa
60S ribosomal protein L6	ribosome component	RL6_HUMAN	646483	Q02878	33 kDa
Death-inducer obliterator 1	weakly pro-apoptotic when overexpressed	DIDO1_HUMAN	11083	Q9BTC0	244 kDa
Serine/arginine-rich splicing factor 3	RNA processing in relation with cellular proliferati	SRSF3_HUMAN	6428	P84103	19 kDa
Profilin-1	actin cytoskeleton organization	PROF1_HUMAN	5216	P07737	15 kDa
RNA-binding protein 14	rna binding	RBM14_HUMAN	5936	Q96PK6	69 kDa
Cleavage and polyadenylation specificity factor subunit	rna splicing	CPSF7_HUMAN	79869	Q8N684	52 kDa
Myosin-9	role in cytokinesis	MYH9_HUMAN	4627	P35579	227 kDa
Vesicular integral-membrane protein VIP36	secretory pathway	LMAN2_HUMAN	10960	Q12907	40 kDa
60S ribosomal protein L10	ribosome component	RL10_HUMAN	285176	P27635	25 kDa
F-actin-capping protein subunit beta	actin cytoskeleton organization	CAPZB_HUMAN	832	P47756	31 kDa
Tight junction protein ZO-1	cell junctions	ZO1_HUMAN	7082	Q07157	195 kDa
Serrate RNA effector molecule homolog	Cell Proliferation/Cell cycle progression S Phase	SRRT_HUMAN	51593	Q9BXP5	101 kDa
Eukaryotic translation initiation factor 2 subunit 1	protein synthesis	IF2A_HUMAN	1965	P05198	36 kDa
KH domain-containing, RNA-binding, signal transduction-a	G2/M transition cell cycle arrest	KHDR1_HUMAN	10657	Q07666	48 kDa
SH3KBP1-binding protein 1		SHKB1_HUMAN	92799	Q8TBC3	76 kDa
CCR4-NOT transcription complex subunit 2	transcriptional regulation	CNOT2_HUMAN	4848	Q9NZN8	60 kDa
U6 snRNA-associated Sm-like protein LSm2	splicasome	LSM2_HUMAN	57819	Q9Y333	11 kDa
TGF-beta-activated kinase 1 and MAP3K7-binding protein 2	activation of MAPK activity	TAB2_HUMAN	23118	Q9NYJ8	76 kDa
60S ribosomal protein L7	poly(A) RNA binding	RL7_HUMAN	648000	P18124	29 kDa
Dedicator of cytokinesis protein 7	microtubule cytoskeleton organization	DOCK7_HUMAN	85440	Q96N67	243 kDa
Zinc finger RNA-binding protein	nucleotide binding	ZFR_HUMAN	51663	Q96KR1	117 kDa
Activating signal cointegrator 1 complex subunit 3	DNA repair cell proliferation	ASCC3_HUMAN	10973	Q8N3C0	251 kDa
RNA-binding protein 10	poly(A) RNA binding	RBM10_HUMAN	8241	P98175	104 kDa
Tetratricopeptide repeat protein 28	condensation of spindle midzone microtubules	TTC28_HUMAN	729515	Q96AY4	271 kDa
Procollagen-lysine,2-oxoglutarate 5-dioxygenase 1	forms hydroxylyisnes in collagens	PLOD1_HUMAN	5351	Q02809	84 kDa
Transformer-2 protein homolog alpha	sequence specific RNA binding	TRA2A_HUMAN	29896	Q13595	33 kDa
Non-POU domain-containing octamer-binding protein	DNA repair pathway	NONO_HUMAN	4841	Q15233	54 kDa
Zinc finger CCHC domain-containing protein 8	pre-mRNA splicing	ZCHC8_HUMAN	55596	Q6NZY4	79 kDa
ELAV-like protein 2	RNA binding	ELAV2_HUMAN	1993	Q12926	40 kDa
Putative helicase MOV-10	nucleotide binding	MOV10_HUMAN	4343	Q9HCE1	114 kDa
A-kinase anchor protein 8	compartmentalization of PKA type II proteins	AKAP8_HUMAN	10270	O43823	76 kDa
Splicing factor, proline- and glutamine-rich	DNA repair pathway	SFPQ_HUMAN	6421	P23246	76 kDa
Programmed cell death protein 6	fas/fadd death pathway	PDCD6_HUMAN	57491	O75340	22 kDa
Dedicator of cytokinesis protein 8	proliferation	DOCK8_HUMAN	81704	Q8NF50	239 kDa
Transformer-2 protein homolog beta	mrna splicing	TRA2B_HUMAN	6434	P62995	34 kDa
Acetyl-CoA carboxylase 1	metabolism	ACACA_HUMAN	31	Q13085	266 kDa
Ubiquitin carboxyl-terminal hydrolase 34	activator of Wnt signaling pathway	UBP34_HUMAN	9736	Q70CQ2	404 kDa
Calponin-3	cytoskeletal organization	CNN3_HUMAN	1266	Q15417	36 kDa
Mediator of RNA polymerase II transcription subunit 12	Chromatin Binding	MED12_HUMAN	9968	Q93074	243 kDa
La-related protein 1	rna binding	LARP1_HUMAN	23367	Q6PKG0	124 kDa
Protein argonaute-2	RNA-mediated gene silencing	AGO2_HUMAN	27161	Q9UKV8	97 kDa
Fragile X mental retardation syndrome-related protein 2	rna binding	FXR2_HUMAN	9513	P51116	74 kDa
Protein transport protein Sec24A	vesicle coating	SC24A_HUMAN	10802	O95486	120 kDa
NHP2-like protein 1	rna binding	NH2L1_HUMAN	4809	P55769	14 kDa
Annexin	Ca+ phospholipid binding	B2R657_HUMAN	310	B2R657 (+4)	53 kDa
60S ribosomal protein L13	ribosome component	RL13_HUMAN	6137	P26373	24 kDa
Midasin	nuclear chaperone	MDN1_HUMAN	23195	Q9NU22	633 kDa
Protein PRRC2A	pre-mRNA splicing	PRC2A_HUMAN	7916	P48634	229 kDa
Stomatin-like protein 2	cardiolipin binding	STML2_HUMAN	30968	Q9UJZ1	39 kDa
Zinc finger CCHC domain-containing protein 3	zinc finger protein	ZCHC3_HUMAN	85364	Q9NUD5	44 kDa
Actin, cytoplasmic 1	cytoskeleton oranization	ACTB_HUMAN	60	P60709 (+1)	42 kDa
Nuclear pore complex protein Nup205	nuclear pore complex mitotic nuclear envelope disass	NU205_HUMAN	23165	Q92621	228 kDa
Eukaryotic translation initiation factor 4 gamma 3	translation initiator	IF4G3_HUMAN	8672	O43432	177 kDa
Protein transport protein Sec24B	vesicle coating	SC24B_HUMAN	10427	O95487	137 kDa
Heterogeneous nuclear ribonucleoprotein H3	nucleotide binding	HNRH3_HUMAN	3189	P31942	37 kDa
Serine hydroxymethyltransferase, mitochondrial	mtDNA	GLYM_HUMAN	6472	P34897	56 kDa
Casein kinase II subunit beta	participates in Wnt Signaling	CSK2B_HUMAN	1460	P67870 (+1)	25 kDa
ATP synthase subunit alpha, mitochondrial	ox/phos	ATPA_HUMAN	498	P25705	60 kDa
Protein transport protein Sec23A	vesicle coating	SC23A_HUMAN	10484	Q15436	86 kDa
Plectin	apoptosis	PLEC_HUMAN	5339	Q15149	532 kDa
YTH domain family protein 2	poly(A) RNA binding	YTHD2_HUMAN	51441	Q9Y5A9	62 kDa
Putative ribosomal RNA methyltransferase NOP2	regulation of cell cycle	NOP2_HUMAN	4839	P46087	89 kDa
Serine/arginine-rich splicing factor 7	pre-mRNA splicing	SRSF7_HUMAN	6432	Q16629	27 kDa
Ubiquitin carboxyl-terminal hydrolase 24	protease	UBP24_HUMAN	23358	Q9UPU5	294 kDa
Neurofibromin	Stimulates the GTPase activity of Ras.	NF1_HUMAN	4763	P21359	319 kDa
Serine/arginine-rich splicing factor 9	pre-mRNA splicing	SRSF9_HUMAN	8683	Q13242	26 kDa

Poly(U)-binding-splicing factor PUF60	DNA binding apoptosis	PUF60_HUMAN	22827	Q9UHX1	60 kDa
Heterogeneous nuclear ribonucleoprotein H2	nucleotide binding	HNRH2_HUMAN	6173	P55795	49 kDa
Nuclear pore complex protein Nup93	nuclear pore complex (NPC) assembly	NUP93_HUMAN	9688	Q8N1F7	93 kDa
Novel protein		Q5VW09_HUMAN	23078	Q5VW09	54 kDa
Class IVb beta tubulin	cytoskeletal organization	Q8IWP6_HUMAN	10383	Q8IWP6	50 kDa
Centrosomal protein of 85 kDa	centrosome component spindle pole	CEP85_HUMAN	64793	Q6P2H3	86 kDa
Transitional endoplasmic reticulum ATPase	fragmentation of Golgi stacks during mitosis	TERA_HUMAN	7415	P55072	89 kDa
Peroxiredoxin-4	redox regulation	PRDX4_HUMAN	10549	Q13162	31 kDa
Probable ATP-dependent RNA helicase DHX36	telomere maintenance	DHX36_HUMAN	170506	Q9H2U1	115 kDa
Small nuclear ribonucleoprotein F	RNA splicing	RUXF_HUMAN	6636	P62306	10 kDa
Insulin-like growth factor 2 mRNA-binding protein 2	mRNA Binding	IF2B2_HUMAN	10644	Q9Y6M1	66 kDa
Eukaryotic translation initiation factor 4 gamma 2	Cell Cycle arrest/Translation initiation	IF4G2_HUMAN	1982	P78344	102 kDa
Interferon regulatory factor 2-binding protein 1	transcriptional co-repressor	I2BP1_HUMAN	26145	Q8IU81	62 kDa
Eukaryotic translation initiation factor 3 subunit B	ribosomal subunit assembly	EIF3B_HUMAN	8662	P55884	92 kDa
Calcium/calmodulin-dependent protein kinase (CaM kinase)	calmodulin dependent protein kinase activity	D6R938_HUMAN	0	D6R938 (+2)	56 kDa
Protein FAM98B	poly(A) RNA binding	FA98B_HUMAN	283742	Q52LJ0	37 kDa
NF-kappa-B-repressing factor	negative regulator of transcription	NKRF_HUMAN	55922	O15226 (+1)	78 kDa
Nuclear pore membrane glycoprotein 210	nuclear pore assembly mitotic cell cycle	PO210_HUMAN	23225	Q8TEM1	205 kDa
Galectin-3-binding protein	integrin mediated cell-adhesion	LG3BP_HUMAN	3959	Q08380	65 kDa
Exosome complex component MTR3	mRNA processing	EXOS6_HUMAN	118460	Q5RKV6	28 kDa
Pre-mRNA cleavage complex 2 protein Pcf11	pre-mrna cleavage complex II	PCF11_HUMAN	51585	O94913	173 kDa
Vacuolar protein sorting-associated protein 13B	golgi protein trafficking	VP13B_HUMAN	157680	Q7Z7G8	449 kDa
Centrosomal protein of 192 kDa	Required for mitotic centrosome and spindle assembly	E9PF99_HUMAN	0	E9PF99	279 kDa
Pre-mRNA 3'-end-processing factor FIP1	pre-mRNA splicing	FIP1_HUMAN	81608	Q6UN15	67 kDa
Myotubularin-related protein 5	cell death	MTMR5_HUMAN	100133234	O95248	208 kDa
ATP synthase subunit beta, mitochondrial	ox/phos	ATPB_HUMAN	506	P06576	57 kDa
40S ribosomal protein S28	ribosome component	RS28_HUMAN	728453	P62857	8 kDa
Mediator of RNA polymerase II transcription subunit 24	regulates transcription of nearly all RNA pols	MED24_HUMAN	9862	O75448	110 kDa
Eukaryotic translation initiation factor 3 subunit D	translation regulator	EIF3D_HUMAN	8664	O15371	64 kDa
U6 snRNA-associated Sm-like protein LSm6	mRNA degradation	LSM6_HUMAN	11157	P62312	9 kDa
DNA-directed RNA polymerases I, II, and III subunit RPAB	NER DNA repair	RPAB3_HUMAN	5437	P52434	17 kDa
3-hydroxyacyl-CoA dehydrogenase type-2	mitochondrial tRNA maturation	HCD2_HUMAN	3028	Q99714	27 kDa
Cleavage and polyadenylation specificity factor subunit	mRNA Cleavage	CPSF1_HUMAN	29894	Q10570	161 kDa
Transportin-1	nuclear localization binding sequence	TNPO1_HUMAN	3842	Q92973	102 kDa
Serine/arginine-rich splicing factor 2	pre-mRNA splicing	SRSF2_HUMAN	6427	Q01130	25 kDa
Mediator of RNA polymerase II transcription subunit 16	regulates transcription of nearly all RNA pols	MED16_HUMAN	10025	Q9Y2X0	97 kDa
40S ribosomal protein S15a	ribosome component	RS15A_HUMAN	646819	P62244	15 kDa
Eukaryotic initiation factor 4A-III	atp binding	IF4A3_HUMAN	9775	P38919	47 kDa
Ataxin-2	EGFR trafficking	ATX2_HUMAN	6311	Q99700	140 kDa
40S ribosomal protein S16	ribosome component	Q6IPX4_HUMAN	6217	Q6IPX4	17 kDa
Mediator of RNA polymerase II transcription subunit 13	transcription activity	MED13_HUMAN	9969	Q9UHV7	239 kDa
Enhancer of mRNA-decapping protein 4	mRNA degradation	EDC4_HUMAN	23644	Q6P2E9	152 kDa
Translational activator GCN1	regulates GCN2 protein Kinase activity	GCN1L_HUMAN	10985	Q92616	293 kDa
rRNA 2'-O-methyltransferase fibrillarin	histone glutamine methylation	FBRL_HUMAN	2091	P22087	34 kDa
Polypyrimidine tract-binding protein 3	pre-mRNA splicing	PTBP3_HUMAN	9991	O95758	60 kDa
Transcriptional activator protein Pur-alpha	DNA replication initiation	PURA_HUMAN	5813	Q00577 (+1)	35 kDa
DNA-directed RNA polymerase I subunit RPA1	transcriptional regulator	RPA1_HUMAN	25885	O95602	195 kDa
Probable fibrosin-1		FBRS_HUMAN	64319	Q9HAH7	48 kDa
Interferon regulatory factor 2-binding protein 2	Trascriptional co-repressor	I2BP2_HUMAN	359948	Q7Z5L9	61 kDa
Cofactor required for Sp1 transcriptional activation, subunit 3, 130kDa		Q5JWT2_HUMAN	9439	Q5JWT2 (+1)	157 kDa
40S ribosomal protein S21	ribosome component	RS21_HUMAN	6227	P63220	9 kDa
40S ribosomal protein S10	ribosome component	RS10_HUMAN	376693	P46783	19 kDa
Zinc finger CCCH-type antiviral protein 1	nucleotide binding	ZCCHV_HUMAN	56829	Q7Z2W4	101 kDa
Ribosomal protein L27a	ribosome component	Q6NZ52_HUMAN	6157	Q6NZ52	16 kDa
Cleavage and polyadenylation specificity factor subunit	poly(A) RNA binding	CPSF6_HUMAN	11052	Q16630	59 kDa
Vesicle-trafficking protein SEC22b	SNARE protein	SC22B_HUMAN	9554	O75396	25 kDa
Nuclear receptor corepressor 2	promotes chormatin condensation	NCOR2_HUMAN	9612	Q9Y618	275 kDa
NFX1-type zinc finger-containing protein 1		ZNFX1_HUMAN	57169	Q9P2E3	220 kDa
60S ribosomal protein L22	ribosome component	RL22_HUMAN	6146	P35268 (+1)	15 kDa
Tubulin beta-2B chain	cytoskeletal organization	TBB2B_HUMAN	347733	Q9BVA1	50 kDa
tRNA pseudouridine synthase-like 1	tRNA maturation	PUSL1_HUMAN	126789	Q8N0Z8	33 kDa
Trinucleotide repeat-containing gene 6B protein	rna mediated silencing	TNR6B_HUMAN	23112	Q9UPQ9	194 kDa
Protein disulfide-isomerase A6	Inhibits protein aggregation	PDIA6_HUMAN	10130	Q15084	48 kDa
Importin subunit alpha-7	nuclear protein import	IMA7_HUMAN	23633	O60684	60 kDa
Cleavage stimulation factor subunit 2	nucleotide binding	B4DUD5_HUMAN	0	B4DUD5 (+1)	59 kDa
Protein TANC2		TANC2_HUMAN	26115	Q9HCD6	220 kDa
Fibrillin-2	calcium binding	FBN2_HUMAN	2201	P35556	315 kDa
U2 snRNP-associated SURP motif-containing protein		SR140_HUMAN	23350	O15042	118 kDa
Polyadenylate-binding protein 2	nucleotide binding	PABP2_HUMAN	8106	Q86U42	33 kDa
Mediator of RNA polymerase II transcription subunit 30	regulates transcription of nearly all RNA pols	MED30_HUMAN	90390	Q96HR3	20 kDa
SMARCA4 isoform 3	negative regulation of G1/S transition	B1A8Z4_HUMAN	6597	B1A8Z4 (+4)	181 kDa
Glutathione S-transferase omega-1	glutathione transferase activity	GSTO1_HUMAN	9446	P78417	28 kDa
39S ribosomal protein L44, mitochondrial	ribosome component	RM44_HUMAN	65080	Q9H9J2	38 kDa
Multivesicular body subunit 12A	vesicle trafficking	F125A_HUMAN	93343	Q96EY5	29 kDa
Splicing factor U2AF 35 kDa subunit	poly(A) RNA binding	U2AF1_HUMAN	7307	Q01081	28 kDa
Pumilio homolog 1	negatively regulates translation and mRNA stability	PUM1_HUMAN	9698	Q14671	126 kDa
FUS interacting protein (Serine/arginine-rich) 1	nucleotide binding	Q5JRI3_HUMAN	653884	Q5JRI3	22 kDa
Serine/arginine-rich splicing factor 6	poly(A) RNA binding	SRSF6_HUMAN	6431	Q13247	40 kDa
Nucleolysin TIAR	apoptosis	TIAR_HUMAN	7073	Q01085	42 kDa
Mediator of RNA polymerase II transcription subunit 27	transcriptional regulator	MED27_HUMAN	9442	Q6P2C8	35 kDa
Eukaryotic translation initiation factor 2 subunit 2	translational regulator	IF2B_HUMAN	8894	P20042	38 kDa
Nucleolysin TIA-1 isoform p40	poly(A) RNA binding	TIA1_HUMAN	7072	P31483	43 kDa
Myb-binding protein 1A	positive regulation of cell cycle arrest	MBB1A_HUMAN	10514	Q9BQG0	149 kDa
Small nuclear ribonucleoprotein component	ribosome component	Q8IXJ3_HUMAN	9343	Q8IXJ3	95 kDa
Pyrroline-5-carboxylate reductase 2	proline biosynthesis	P5CR2_HUMAN	29920	Q96C36	34 kDa
F-box-like/WD repeat-containing protein TBL1XR1	Wnt Signaling pathway	TBL1R_HUMAN	79718	Q9BZK7	56 kDa
Protein SCAF8	mRNA processing	SCAF8_HUMAN	22828	Q9UPN6	141 kDa
LanC-like protein 1	binds glutathione	LANC1_HUMAN	10314	O43813	45 kDa
Casein kinase I isoform alpha	atp binding	E7ETM0_HUMAN	0	E7ETM0	31 kDa
Myosin-10	cytokenesis	MYH10_HUMAN	4628	P35580	229 kDa
Isocitrate dehydrogenase [NAD] subunit alpha, mitochondr	ox/phos	IDH3A_HUMAN	3419	P50213	40 kDa
Putative RNA-binding protein 3	rna binding	RBM3_HUMAN	5935	P98179	17 kDa
28S ribosomal protein S18b, mitochondrial	ribosome component	RT18B_HUMAN	28973	Q9Y676	29 kDa
Heat shock protein HSP 90-alpha	molecular chaperone involved in cell cycle	HS90A_HUMAN	3320	P07900	85 kDa
U2 small nuclear ribonucleoprotein B''	pre-mRNA splicing	RU2B_HUMAN	6629	P08579	25 kDa
Serine-protein kinase ATM	hub protein	ATM_HUMAN	651610	Q13315	351 kDa
Cation-independent mannose-6-phosphate receptor	transport of lysozomal enzymes	MPRI_HUMAN	3482	P11717	274 kDa
X-ray repair cross-complementing protein 6	DSBR	XRCC6_HUMAN	389901	P12956	70 kDa
182 kDa tankyrase-1-binding protein		TB182_HUMAN	85456	Q9C0C2	182 kDa
Arf-GAP domain and FG repeat-containing protein 1	vesicle fusion	E9PHX7_HUMAN	0	E9PHX7 (+1)	61 kDa
Peroxisomal proliferator-activated receptor A-interactin	atp binding	PR285_HUMAN	85441	Q9BYK8	295 kDa
RNA-binding protein 4	rna binding	RBM4_HUMAN	5936	Q9BWF3	40 kDa
39S ribosomal protein L22, mitochondrial	ribosome component	E7ESL0_HUMAN	0	E7ESL0 (+1)	24 kDa
Ras GTPase-activating-like protein IQGAP1	binds cdc42 cytoskeleton remodeling	IQGA1_HUMAN	8826	P46940	189 kDa
Integrator complex subunit 1	negative regulator of apoptosis	INT1_HUMAN	26173	Q8N201	244 kDa
Syntaxin-7	protein trafficking	STX7_HUMAN	8417	O15400	30 kDa
Protein lin-7 homolog C	exocytosis	LIN7C_HUMAN	55327	Q9NUP9	22 kDa
39S ribosomal protein L49, mitochondrial	ribosome component	RM49_HUMAN	740	Q13405	19 kDa
Transcription elongation factor SPT5	cell cycle	SPT5H_HUMAN	6829	O00267	121 kDa
Nuclear cap-binding protein subunit 1	g-cap on mrna	NCBP1_HUMAN	4686	Q09161	92 kDa
Probable tRNA pseudouridine synthase 2		TRUB2_HUMAN	26995	O95900	37 kDa
Double-stranded RNA-specific adenosine deaminase	nucleotide binding	DSRAD_HUMAN	103	P55265	136 kDa
Transformation/transcription domain-associated protein	chromatin organization	TRRAP_HUMAN	8295	Q9Y4A5	438 kDa

Protein KIAA1967	genome stability after UV stress	K1967_HUMAN	57805	Q8N163	103 kDa
BAG family molecular chaperone regulator 4	inhibits hsp70 activity	BAG4_HUMAN	9530	O95429	50 kDa
60S ribosomal protein L18a	ribosome component	B2R4C0_HUMAN	0	B2R4C0	21 kDa
Protein TANC1	scaffold component	TANC1_HUMAN	85461	Q9C0D5	202 kDa
Keratin, type I cytoskeletal 19		K1C19_HUMAN	3880	P08727	44 kDa
Epiplakin	poly(A) RNA binding	EPIPL_HUMAN	83481	P58107	556 kDa
SRSF protein kinase 1	chromatin reorganization in the cell cycle	SRPK1_HUMAN	6732	Q96SB4	74 kDa
Laminin alpha 3 splice variant b2	cell adhesion	Q6VU67_HUMAN	3909	Q6VU67	360 kDa
Partitioning defective 3 homolog	asymetrical cell division and cell polarization	PARD3_HUMAN	56288	Q8TEW0	151 kDa
Tubulin beta-6 chain	cytoskeleton component	TBB6_HUMAN	84617	Q9BUF5	50 kDa
Serine/arginine-rich splicing factor 5	rna splicing regulator of cell cycle	SRSF5_HUMAN	6430	Q13243	31 kDa
Peflin	calcium binding	PEF1_HUMAN	553115	Q9UBV8	30 kDa
Replication protein A 70 kDa DNA-binding subunit	dna repair and G1/S transition also mitosis	RFA1_HUMAN	6117	P27694	68 kDa
Triple functional domain protein	gdp-gtp exchanger	TRIO_HUMAN	7204	O75962	347 kDa
Prohibitin-2	mitochondrial respiration activity	PHB2_HUMAN	11331	Q99623	33 kDa
Structural maintenance of chromosomes flexible hinge dom	required for DUX4 silencing (G0/G1 transition)	SMHD1_HUMAN	23347	A6NHR9	226 kDa
Single-stranded DNA-binding protein 3	cell proliferation	SSBP3_HUMAN	23648	Q9BWW4	40 kDa
Dedicator of cytokinesis protein 6	exchange factor for cdc42 and rac1	DOCK6_HUMAN	57572	Q96HP0	230 kDa
Cysteine and glycine-rich protein 2	zinc ion binding	F8VW96_HUMAN	0	F8VW96 (+1)	27 kDa
Keratin, type I cytoskeletal 18		K1C18_HUMAN	3875	P05783	48 kDa
AT-rich interactive domain-containing protein 1A	atp dependent DNA binding	ARI1A_HUMAN	8289	O14497	242 kDa
Replication factor C subunit 5	s phase and mitotic cell cycle	RFC5_HUMAN	5985	P40937	38 kDa
Talin-1	integrin binding	TLN1_HUMAN	7094	Q9Y490	270 kDa
60S ribosomal protein L35a	ribosome component	RL35A_HUMAN	6165	P18077	13 kDa
60S ribosomal protein L36	ribosome component	RL36_HUMAN	347292	Q9Y3U8	12 kDa
Collagen alpha-1(XII) chain		D6RGG3_HUMAN	0	D6RGG3 (+1)	333 kDa
Thyroid hormone receptor-associated protein 3	pre-mRNA splicing	TR150_HUMAN	9967	Q9Y2W1	109 kDa
Cleavage stimulation factor subunit 1	cleavage of pre-mRNAs	CSTF1_HUMAN	1477	Q05048	48 kDa
Laminin subunit gamma-1	ecm constituent	LAMC1_HUMAN	3915	P11047	178 kDa
Zinc finger CCCH domain-containing protein 4	zinc ion binding	ZC3H4_HUMAN	23211	Q9UPT8	140 kDa
Annexin	calcium dependent lipid binding	B4DPJ2_HUMAN	0	B4DPJ2 (+2)	46 kDa
Proteasome subunit beta type-6	DDR and G1/S transition	PSB6_HUMAN	5694	P28072	25 kDa
CDKN2AIP N-terminal-like protein		C2AIL_HUMAN	91368	Q96HQ2	13 kDa
PDZ domain-containing protein GIPC1	G-protein linked signaling	GIPC1_HUMAN	10755	O14908	36 kDa
Growth arrest and DNA damage-inducible proteins-interact	negative regulator of cell cycle G1/S progression	G45IP_HUMAN	90480	Q8TAE8	25 kDa

END OF FILE

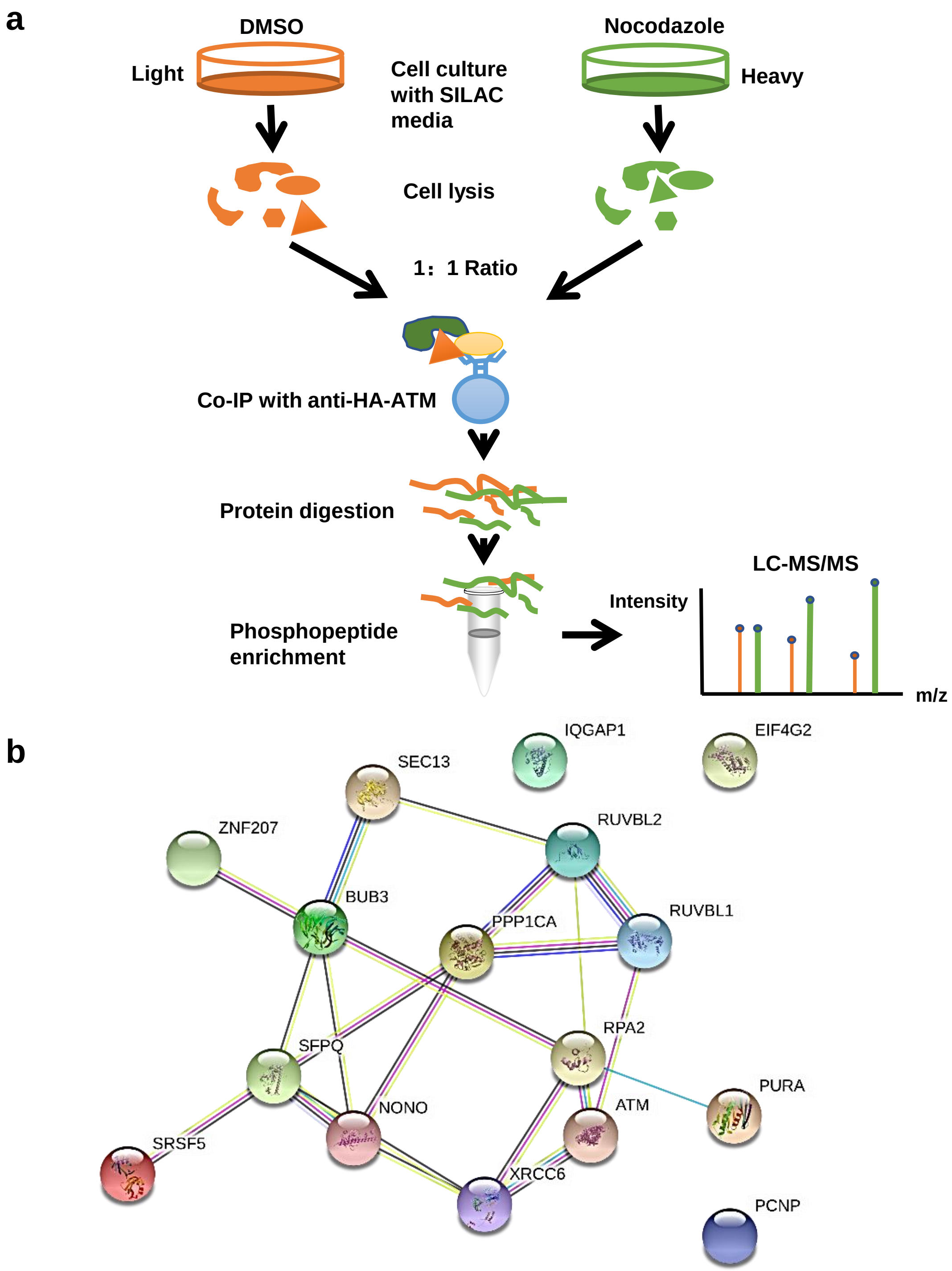

Supplementary Figure S1. ATM substrates in mitosis. **a** The schematic model of the SILAC assay conducted to identify ATM substrates in mitosis. **b** Proteins associated with cell cycle regulation and the DDR.

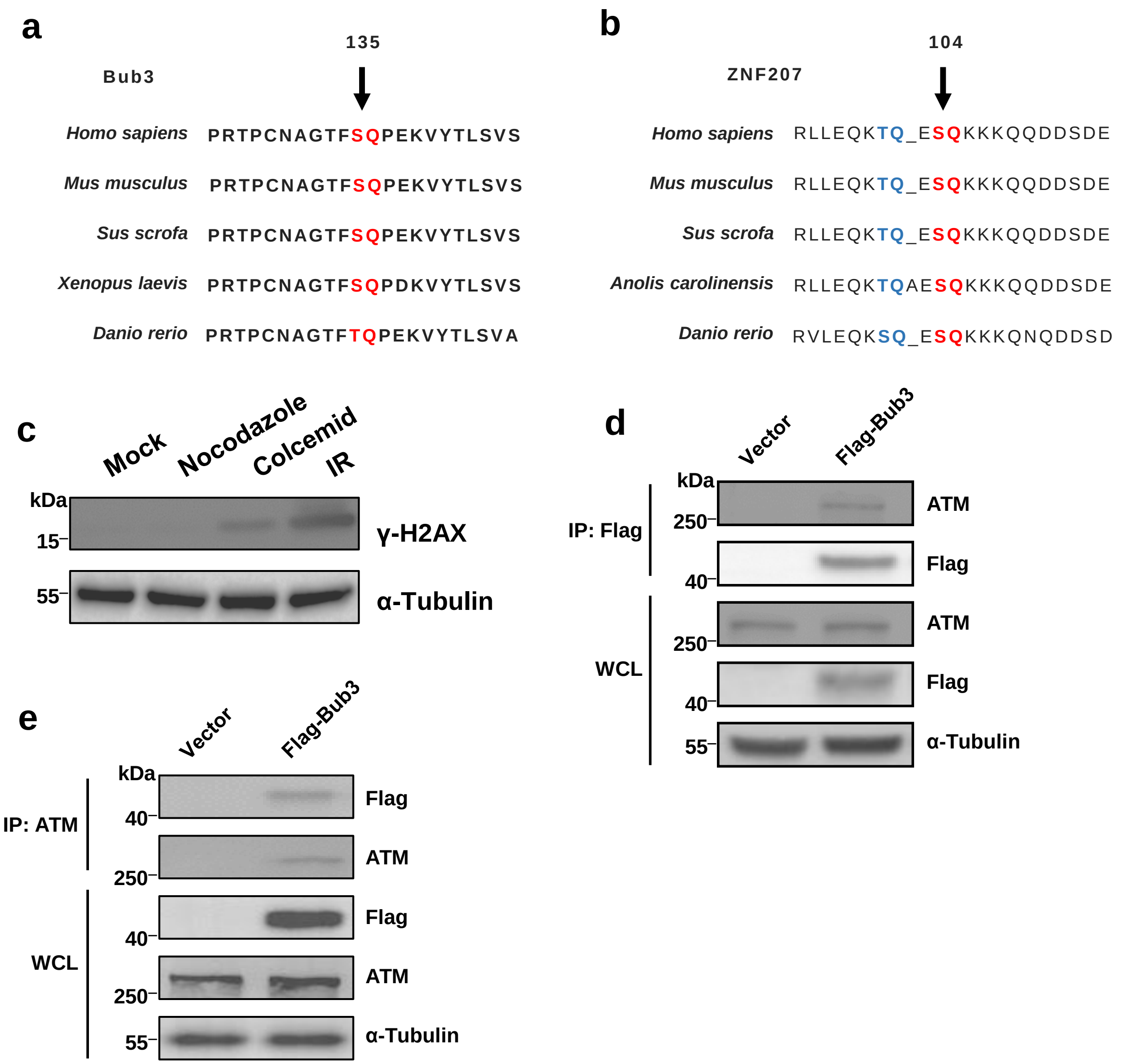

Supplementary Figure S2. a,b Conservation analysis of the Bub3 and ZNF207 sequences identified by SILAC. **c** Whole cell lysates were collected from HeLa cells treated with mock, colcemid (20ng/ml,17h), nocodazole (200nM,17h), or IR (2Gy, 2hr), followed by Western blot using indicated antibodies. **d, e** HeLa cells transfected with vector or Flag-Bub3 were immunoprecipitated with an anti-Flag antibody and analyzed by Western blot using indicated antibodies.

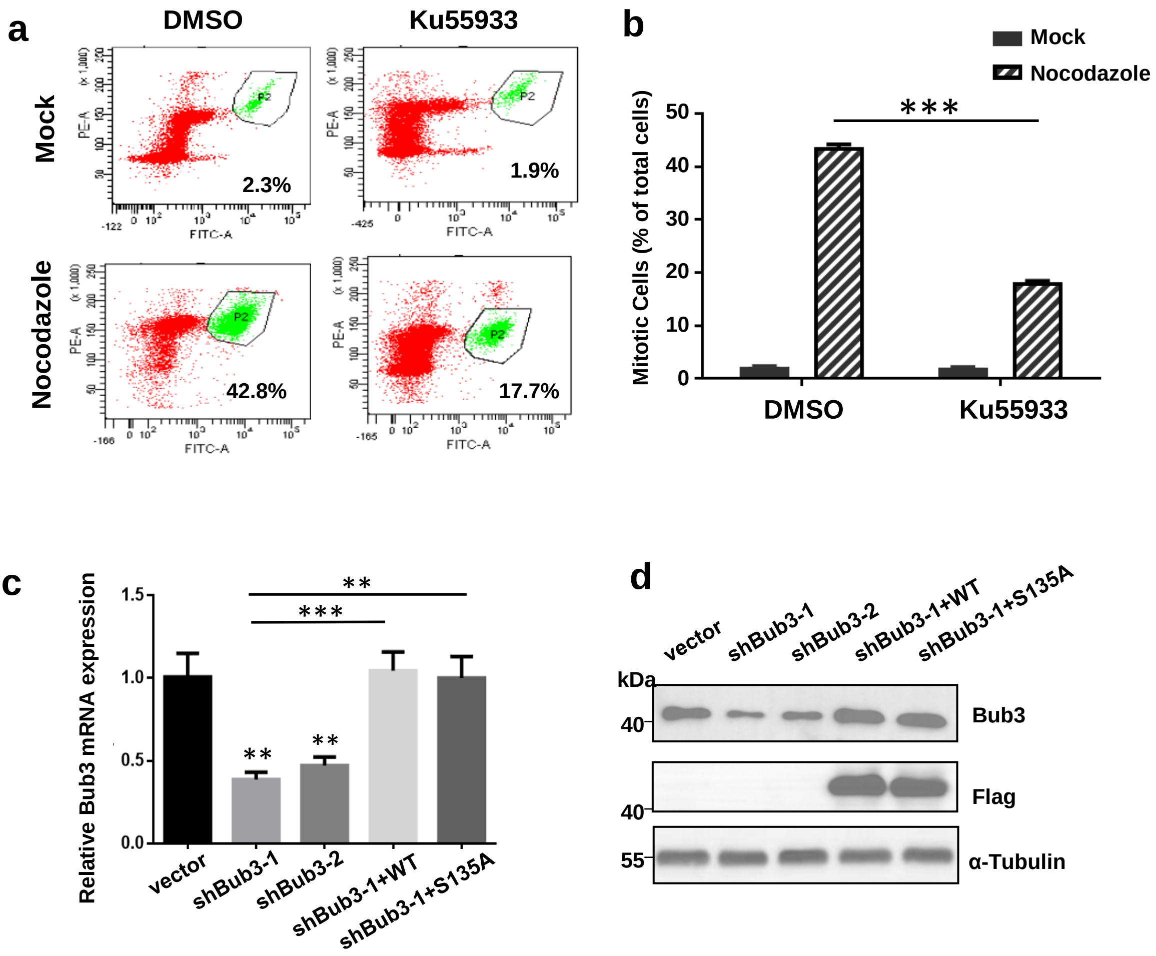

Supplementary Figure S3. a, b Cell cycle analysis and quantification. HeLa cells were treated with nocodazole for 17h in the absence or presence of the ATM inhibitor KU55933. They were stained with a flow cytometry-based anti-phosphor-histone-H3-Ser10 antibody to determine the mitotic index. **c, d** Construction and validation of the isogenic HeLa cell lines stably knocking down Bub3 (shBub3-1, and shBub3-2) and reconstitution of the cells with the wild-type(WT) or S135A mutant Bub3. RT-qPCR assay (**c**) and Western bolt assay (**d**) were carried out to measure Bub3 expression.

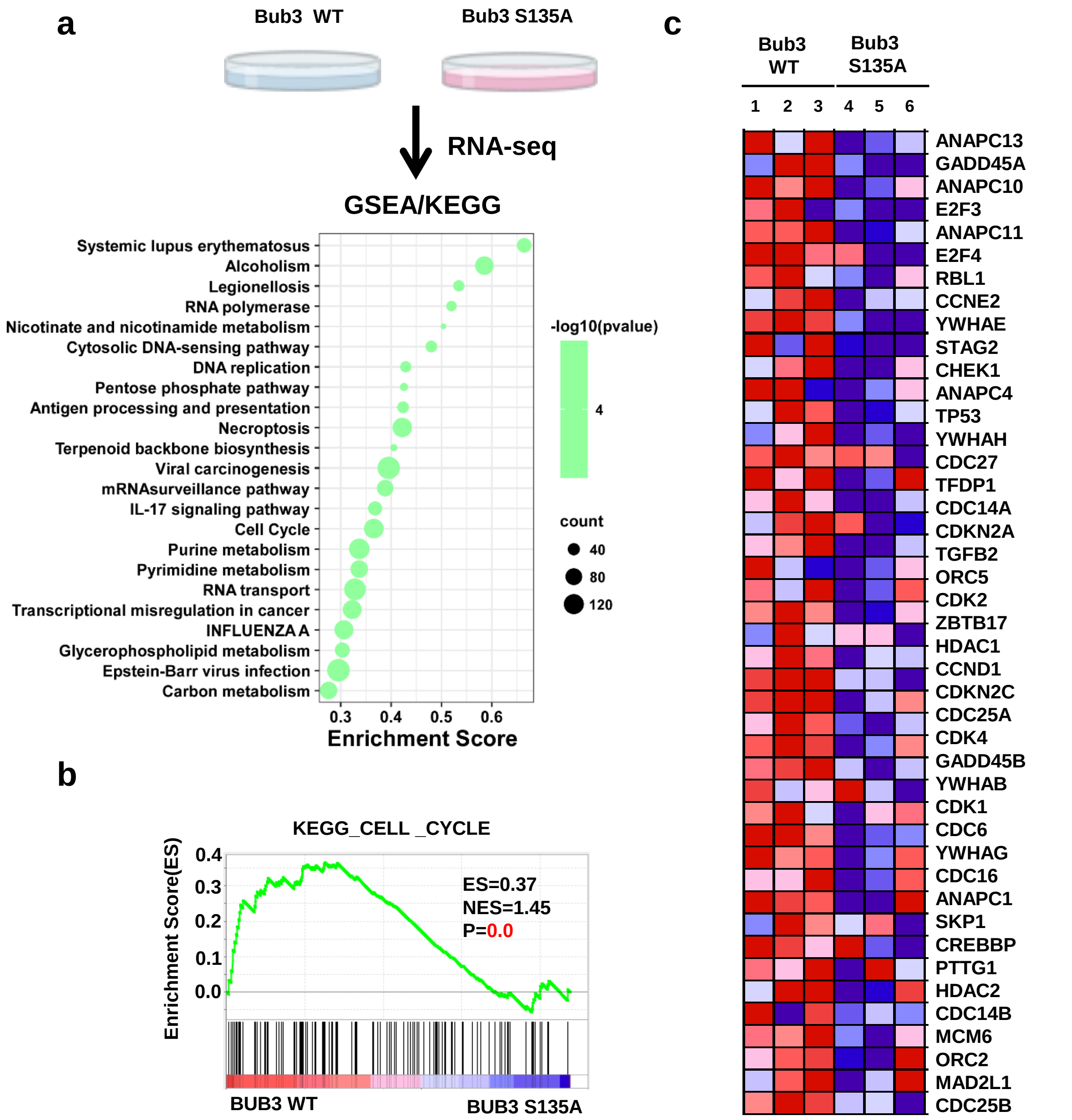

Supplementary Figure S4. RNAseq analysis in HeLa cells expressing WT or S135A Bub3. **a** Signaling pathways enriched in the WT group are shown. **b,c** The GSEA analysis of the correlation between the S135A mutation and the KEGG_cell_cycle gene signature.

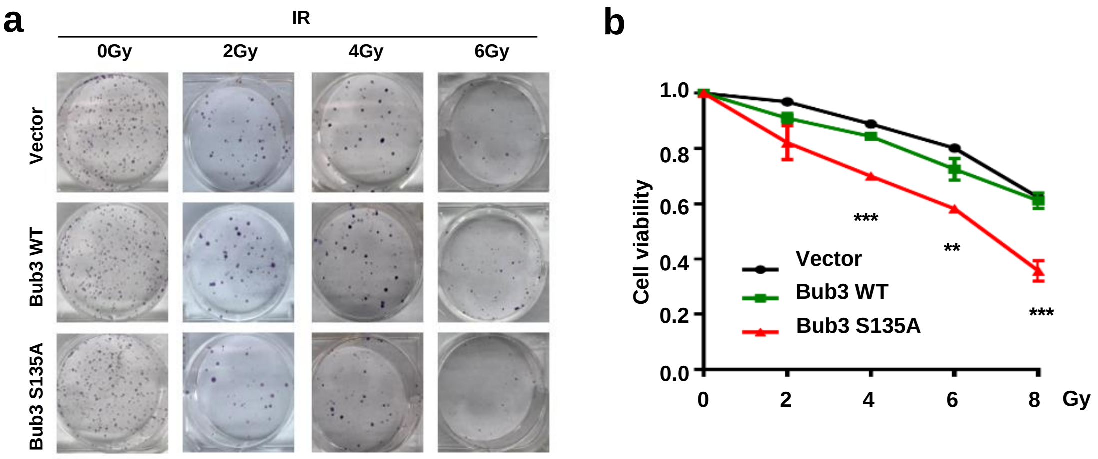

Supplementary Figure S5. a Radiosensitivity was measured with the colony formation assay. **b** Cells expressing the vector, Flag-Bub3 WT, or Flag-Bub3 S135A were irradiated and assessed via MTT assay.

a

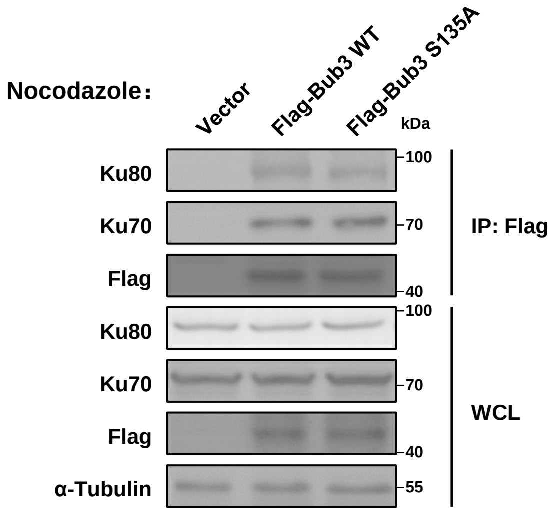

Supplementary Figure S6. a HeLa cells transfected with vector, Flag-Bub3 WT or Flag-Bub3 S135A were treated with mock or nocodazole. Immunoprecipitation of Flag-tagged proteins were conducted followed by Western blot using the indicated antibodies.
